# TET2 has endothelial-specific roles in interferon responses that are dysregulated by hyperglycemia *in vitro* and *in vivo*

**DOI:** 10.1016/j.jbc.2025.110520

**Published:** 2025-07-25

**Authors:** Hannah L.H. Green, Hashum Sum, Palak Sinha, Asjad Visnagri, Sang-Hyuck Lee, Anastasia Baffour-Kyei, Hyunah Lee, Francisco Santos, Konstantinos Theofilatos, Alison C. Brewer

**Affiliations:** 1 School of Cardiovascular and Metabolic Medicine & Sciences, British Heart Foundation Centre of Research Excellence, King’s College London, London, UK; 2 Institute of Psychiatry, Psychology and Neuroscience, King’s College London, London, UK

**Keywords:** endothelial dysfunction, endothelial cell, epigenetics, interferon, TET2, diabetes, DNA methylation, nanopore sequencing

## Abstract

Dysregulated DNA methylation in the endothelium is associated with the development of cardiovascular disease. Ten-eleven translocation 2 (TET2), a DNA demethylase, plays a regulatory role in endothelial function. Loss of endothelial-expressed TET2 correlates with atherosclerosis progression *in vivo* and alters vasoactive signaling *in vitro*. Hyperglycemia acts to downregulate TET2 stability and activity in endothelial cells, but the relevance of this to endothelial dysfunction in diabetes remains unclear. Here, we explore the transcriptional and functional consequences of endothelial-specific TET2 loss *in vivo* and assess the role of DNA methylation in TET2-dependent transcriptional regulation. *Ex vivo* aortic responses to acetylcholine and phenylephrine were equivalent between wild-type and endothelial-specific TET2 knockout (TET2KO) mice. RNA sequencing of endothelial-enriched lung cells from TET2KO mice revealed significant dysregulation of interferon signaling. In cultured endothelial cells, qPCR, hydroxymethylated-DNA immunoprecipitation sequencing, and nanopore sequencing showed that IFITM1 and ISG15—classical interferon-responsive genes—were negatively regulated by TET2 independent of DNA demethylation. Conversely, the IFNγ-inducible chemokines CXCL9 and CXCL10 were positively regulated by TET2 in a mechanism involving catalytic demethylation, as evidenced by increased 5hmC and decreased 5 mC at an enhancer region. Strikingly, approximately 70% of transcriptional changes observed in TET2KO endothelium were mirrored in diabetic mouse endothelium. Pathway analysis highlighted dysregulation of interferon signaling and altered glycosaminoglycan metabolism as the most significant biological consequences. In summary, endothelial TET2 loss leads to transcriptional dysregulation of interferon-regulated genes, partly through altered DNA methylation. This may have relevance to the susceptibility of diabetics to recurrent viral infections and endothelial dysfunction.

Endothelial dysfunction is considered the initiating event in the pathogenesis of vascular disease and is an independent predictor of future cardiovascular events (1, 2, 3). Endothelial dysfunction encompasses an imbalance of endothelial cell (EC) production of vasodilators and vasoconstrictors, expression of leukocyte and platelet adhesion molecules on the EC surface, release of pro-inflammatory cytokines, altered angiogenic responses, and compromised barrier function (4, 5). Diabetes, in particular type 2 diabetes, is now a global pandemic (6, 7), and for individuals with diabetes, cardiovascular diseases (CVDs) are the leading causes of morbidity and mortality (8). The hyperglycemia associated with diabetes is an independent risk factor for CVD, and it is well established that high glucose exposure can act to activate and dysregulate the endothelium. (9, 10). However, despite recent advances, the precise mechanisms by which endothelial dysfunction is initiated and perpetuated in vascular disease and, in particular, in the diabetic setting, remain incompletely understood.

Epigenetic modifications, most notably DNA methylation, are known to elicit dynamic alterations in transcription in response to changes in the cellular environment and are heritable by daughter cells, enabling persistent changes in cell phenotype (11). Many studies have highlighted an association between altered patterns of DNA methylation and endothelial dysfunction in cardiovascular disease, including that associated with hyperglycemic conditions (12, 13, 14, 15, 16). In addition, the administration of high glucose to ECs *in vitro* has, *per se*, been shown to result in aberrant DNA methylation and corresponding altered expression of genes associated with metabolic and cardiovascular disease (15). Thus, it is suggested that the dysregulation of DNA methylation within the endothelium by high glucose exposure may, at least in part, be causal in diabetes-associated vascular disease. Clearly, therefore, the epigenetic modifiers that determine genomic DNA methylation patterns in this context may represent attractive therapeutic targets.

Active DNA demethylation within mammalian cells is mediated by the TET (Ten-Eleven Translocation) family of enzymes, comprised of the methylcytosine dioxygenases TET1, TET2, and TET3, which catalyze the oxidation of 5-methylcytosine (5mC) to 5-hydroxymethylcytosine (5hmC) and subsequently 5-formylcytosine (5fC) and 5-carboxylcytosine (5caC) (17, 18). Unmethylated cytosine is restored either by passive loss during DNA replication or active removal of the oxidative derivatives by base excision repair machinery (17). Previously identified as a tumor suppressor gene, TET2, specifically, has gained interest in the cardiovascular field in recent years with the finding that TET2 loss-of-function mutations are amongst the most common mutations in clonal hematopoiesis of indeterminate potential (CHIP), referring to the benign clonal expansion of hematopoietic cells (19, 20). Further, the presence of CHIP, harboring mutations in TET2, is associated with an approximately twofold increased risk of coronary heart disease (20) and a 25% increased risk of heart failure (21). The causal effect of TET2 loss-of-function in clonally expanded hematopoietic cells on the development of atherosclerosis has been demonstrated robustly in atherosclerosis-prone mice and is understood to involve excess interleukin-1 beta (IL-1β) and IL-6 secretion by TET2-deficient macrophages (20, 22, 23). However, the involvement of the catalytic demethylation activity of TET2, and the genomic loci directly targeted by this activity were not demonstrated or identified in these studies.

Within ECs, TET2 has emerged as the most functionally relevant of the TET family members (24, 25, 26), although its role in vascular disease is not well understood. Nonetheless, previous studies have reported a decrease in TET2 expression (and 5hmC levels) in human atherosclerotic plaques (27) and in the aorta during atherosclerotic lesion progression in an ApoE^−/−^ high-fat diet mouse model, which appears, at least in part, to affect the intimal layer of the vessel wall (25, 26). Athero-prone regions of the vascular tree are associated with low and/or turbulent shear stress, and significantly, TET2 was shown to be downregulated in ECs *in vitro*, in studies conducted to model the low shear stresses encountered in such regions. Furthermore, under these conditions of low shear stress, siRNA-mediated silencing of TET2 led to the downregulation of endothelial nitric oxide synthase (eNOS) and upregulation of endothelin-1, while overexpression of TET2 conversely had the opposite effect (25). There is also evidence to suggest that TET2 regulates the cystathionine γ-lyase/hydrogen sulfide (CSE/H_2_S) system in ECs exposed to oxidized low-density lipoprotein (LDL), by upregulating CSE in a manner involving direct TET2-mediated DNA demethylation of the CSE gene promoter (28).

The molecular mechanisms by which TET2 is downregulated by altered shear stress (described above) are not understood. However, in the context of diabetes, it has been clearly demonstrated that TET2 protein stability and activity can be modulated by post-translational 5′ adenosine monophosphate-activated protein kinase (AMPK)-mediated phosphorylation, which is known to be disrupted under hyperglycemic conditions (29). Indeed, peripheral blood mononuclear cells (PBMCs) from diabetic patients displayed decreased TET2 phosphorylation, decreased TET2 expression, and decreased levels of 5hmC (29). This loss of 5hmC could be recapitulated *in vitro* by culturing PBMCs under high-glucose conditions and was also observed in HUVEC exposed to high glucose concentrations (29). However, the impact of the loss of TET2 activity in ECs upon vascular disease in the context of diabetes has not been investigated.

In this study, we investigated the function of TET2 and the potential consequences of its depletion in EC-specific TET2 knockout (KO) mice. The evidence from *in vitro* studies that TET2 regulates the expression of the potent vasoconstrictor endothelin-1 and of the enzymes responsible for the production of vasodilators nitric oxide (NO) and H_2_S (a component of endothelium-derived hyperpolarizing factor) (25, 28) might suggest that EC-expressed TET2 may contribute to the regulation of vascular tone. Furthermore, although some targets of TET2-mediated transcriptional regulation have been identified in ECs, an unbiased transcriptomic analysis of TET2-ablated ECs (which may yield further insight into the role of TET2 in endothelial (dys)function and notably in the context of diabetes associated vascular disease) has yet to be performed. Using *ex vivo* aortic ring preparations from wild-type (WT) and EC-specific TET2 knockout (KO) mice, the first aim of this study was to determine whether endothelial TET2 is a regulator of vascular tone. In addition, using transcriptomic and epigenomic analyses, we aimed to identify downstream transcriptional targets of TET2 activity in ECs, which may contribute to endothelial (function and) dysfunction in vascular disease. We identify transcriptional IFNγ responses to be modulated by TET2 in ECs and to involve both catalytic and non-catalytic mechanisms. Finally, by comparison of the transcriptome of ECs isolated from TET2 KO mice with those from diabetic mice, we identify a striking overlap, suggesting that the loss of TET2 in ECs in diabetes may be a major causal factor in associated CVD.

## Results

### Depletion of TET2 in ECs does not affect aortic vascular reactivity

**Figure 1:**
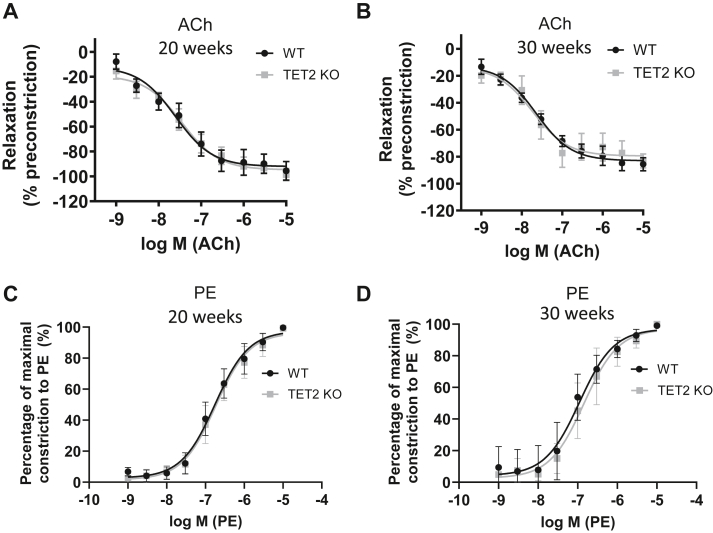
**Aortic responses to ACh and PE are equivalent in WT and EC-specific TET2 KO mice.** Aortic rings were isolated from male TET2^fl/fl^ CDH5-CreERT2 Cre-ve (WT) and Cre+ve (TET2 KO) tamoxifen-injected C57Bl/6J mice and suspended under isometric conditions in organ bath chambers containing Krebs’ buffer. The tension generated by the vessels in response to agonists was recorded. The extent of relaxation of vessels from mice at 20 weeks (*A*) and 30 weeks of age (*B*) following the addition of 10^−9^M and 10^−5^M ACh after preconstruction with 3 × 10^−9^M phenylephrine (PE). The percentage of maximal constriction resulting from 10^−9^M to 10^−5^M PE in vessels from mice at 20 weeks (*C*) and 30 weeks of age (*D*). Data presented as mean ± SEM from n = 5-9 per group.

TET2 depletion in ECs *in vitro* has been demonstrated to alter the expression of eNOS and CSE (25, 28). We therefore tested the effect of EC-specific ablation of TET2 upon the regulation of vascular tone *in vivo*. EC-specific TET2 “knockout” (KO) mice were generated by crossing TET2^fl/fl^ mice (30) to tamoxifen-inducible, EC-specific Cre-expressing mice (Cdh5-CreERT2) (31). Successful Cre-recombination following tamoxifen injection was confirmed (Fig. S1). Aortic rings were isolated from male, tamoxifen-injected Cre^+ve^ TET2^fl/fl^ mice (TET2 KO) and their Cre^-ve^ TET2^fl/fl^ littermates (WT), and vascular reactivity was measured *ex vivo* under isometric conditions using an organ bath system. Vessels were preconstricted with phenylephrine and exposed to increasing concentrations of ACh to measure endothelium-dependent vasorelaxation. No difference in vasorelaxation was observed in response to ACh between WT and TET2 KO animals at 20 weeks of age (Fig. 1*A*). The dose response of phenylephrine-induced contraction was also measured because endothelium-derived vasodilators can modulate the extent of vasoconstriction in response to phenylephrine (as previously shown in our laboratory (32)). However, no difference in the constrictor response to phenylephrine was evident (Fig. 1*C*). Given that endothelial dysfunction develops over time, measurements were repeated in a second cohort of mice at 30 weeks of age (Fig. 1, *B* and *D*). At this time point, ACh and PE responses remained equivalent between WT and TET2 KO mice. A difference in sodium nitroprusside (SNP)-mediated vasorelaxation was not anticipated between WT and TET2 KO mice, as its vasodilator activity is elicited by acting directly on vascular smooth muscle cells, which would not be affected by the EC-specific TET2 deletion. Indeed, no difference in the dose response to SNP was observed in these mice (Figs. S2*B* and S3*B*).

### Transcriptomic analysis of endothelial-enriched cells from murine lungs reveals differential expression of leukocyte activation and IFNγ-signaling pathway genes following TET2 ablation

**Figure 2:**
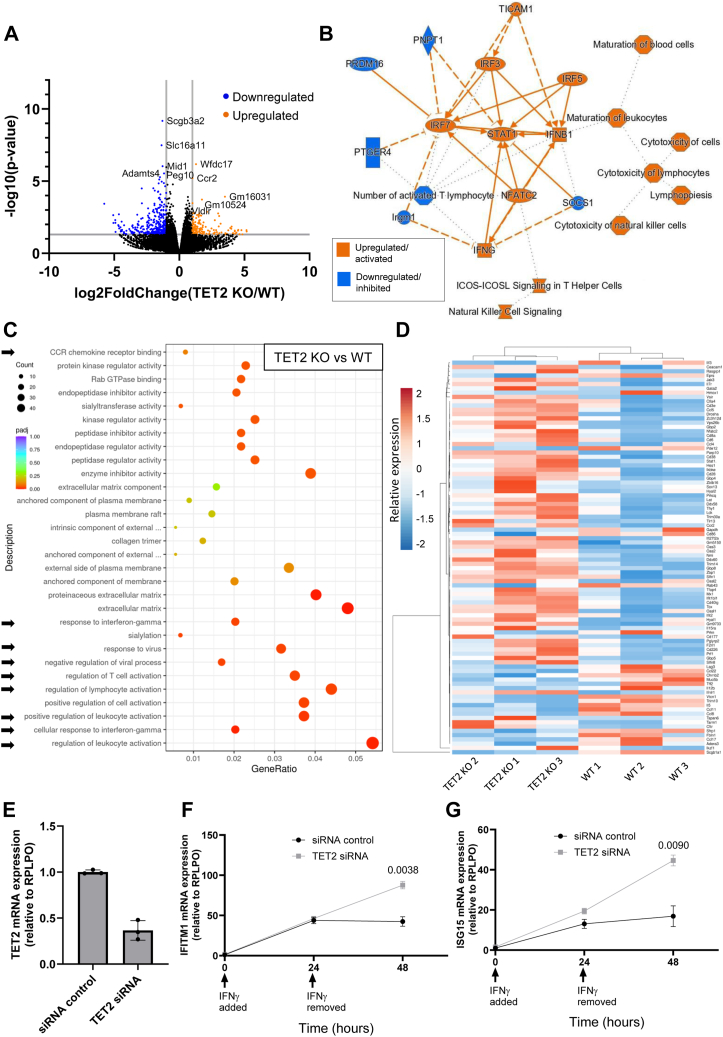
**Differential gene expression in CD31+ cells from lungs of WT and EC-specific TET2 KO mice and expression of IFN-responsive genes in TET2-silenced HUVEC.***A*, Volcano plot displaying transcripts detected by RNA sequencing of WT and TET2 KO CD31+ cells. *Blue* color indicates log2FoldChange > −1 and −log10 (*p*-value) > 1.3, considered significantly downregulated in TET2 KO cells compared to WT cells. *Orange* color indicates log2FoldChange > 1 and −log10 (*p*-value) > 1.3, considered significantly upregulated in TET2 KO cells compared to WT cells. The five genes with the greatest *p-values* are annotated. *B*, ingenuity pathway analysis graphical summary generated using a machine learning algorithm identified the major biological features distinguishing the TET2 KO and WT CD31+ transcriptome. *C*, gene ontology annotation of biological processes, molecular functions, and cellular components with significantly different enrichment in TET2 KO cells compared to WT cells (determined by gene set enrichment analysis). The count of differentially expressed genes (DEGs) associated with each feature is denoted by the size of the circle. Statistical significance is indicated by the color scale. *Arrows* indicate annotations relating to leukocyte activation, viral and IFN responses. *D*, Heatmap and hierarchical clustering showing the relative expression of DEGs belonging to pathways associated with leukocyte activation, viral and IFN responses in three biological replicates of TET2 KO and WT CD31+ cells. E) TET2 mRNA expression of HUVEC transfected with negative control siRNA or siRNA targeting TET2. *F, G*, Relative mRNA expression of IFITM1 (*F*) and ISG15 (*G*) in HUVEC transfected with negative control siRNA or siRNA targeting TET2 and treated with IFNγ for 0 or 24 h o,r treated with IFNγ for 24 h followed by its removal for 24 h. A Shapiro-Wilk test for normality was performed, followed by an unpaired *t* test at 24 h and 48 h timepoints. Data presented as mean ± SEM. n = 3 triplicate samples, representative of three independent experiments.

To determine how TET2 depletion might influence other aspects of endothelial function, we analyzed the transcriptomes of endothelial-enriched (CD31+ cells) isolated from the lungs of WT and TET2 KO mice. Using RNA sequencing, we thus identified, in an unbiased manner, putative TET2-regulated genes in these cells. 495 differentially expressed genes (Log2FoldChange>|1| and *p* < 0.05) were identified, of which 347 were downregulated and 148 were upregulated in TET2 KO mice compared to WT mice (Fig. 2*A*) (data are publicly available *via* the GEO database, deposited under GSE232888). Further transcriptomic analysis was performed using Ingenuity Pathway Analysis (Qiagen) to identify biological pathways and upstream regulators of the differentially expressed genes. A graphical summary of the major features distinguishing WT and TET2 KO cells revealed a convergence of pathways involving upregulation of STAT1, IFNγ, IFNβ1, and multiple interferon (IFN) response factors. A cluster of functions related to maturation and cytotoxicity of leukocytes, including lymphocytes and natural killer cells, was also apparent (Fig. 2*B*). Furthermore, gene ontology annotations similarly mapped to biological pathways associated with leukocyte activation and IFNγ signaling, in addition to pathways involved in membrane anchoring and the extracellular matrix (Fig. 2*C*). Notably, the majority of genes belonging to pathways associated with leukocyte activation and IFNγ signaling were *upregulated* in TET2 KO cells compared to WT cells (Fig. 2*D*).

### Impaired resolution of IFNγ signaling in TET2-silenced HUVEC

We next sought to validate the involvement of TET2 in regulating the IFN response *in vitro* using human ECs. Two well-studied classical IFN-responsive genes expressed by ECs were selected to probe the effect of TET2 silencing on both the activation and subsequent decay of IFN responses: IFN-induced transmembrane protein 1 (IFITM1) and IFN-sensitive gene 15 (ISG15) (33). IFITM1 and ISG15 possess both an IFN-stimulated response element (ISRE) and a gamma-activated site (GAS) in their promoter regions, so their expression can be induced by either type I IFN or type II IFN, although they may do so to varying degrees (34, 35, 36). However, the sole type II IFN, IFNγ, was selected for our study owing to its strong association with the pathogenesis of cardiovascular disease and, in particular, in diabetes (37, 38, 39). In control and TET2-silenced HUVEC (Fig. 2*E*), the mRNA expression of IFITM1 and ISG15 was measured by qPCR at baseline, after 24 h of IFNγ treatment, and 24 h after its removal (Fig. 2, *F* and *G*). The expression of both IFITM1 and ISG15 in TET2-silenced, compared to control, cells was (only slightly) increased 24 h after the addition of IFNγ. However, in marked contrast to the controls, the expression of both genes continued to rise significantly in the 24 h following removal of IFNγ (Fig. 2, *F* and *G*).

### No evidence for involvement of DNA demethylation in IFNγ-activation of IFITM1 or ISG15 transcription in HUVEC

**Figure 3:**
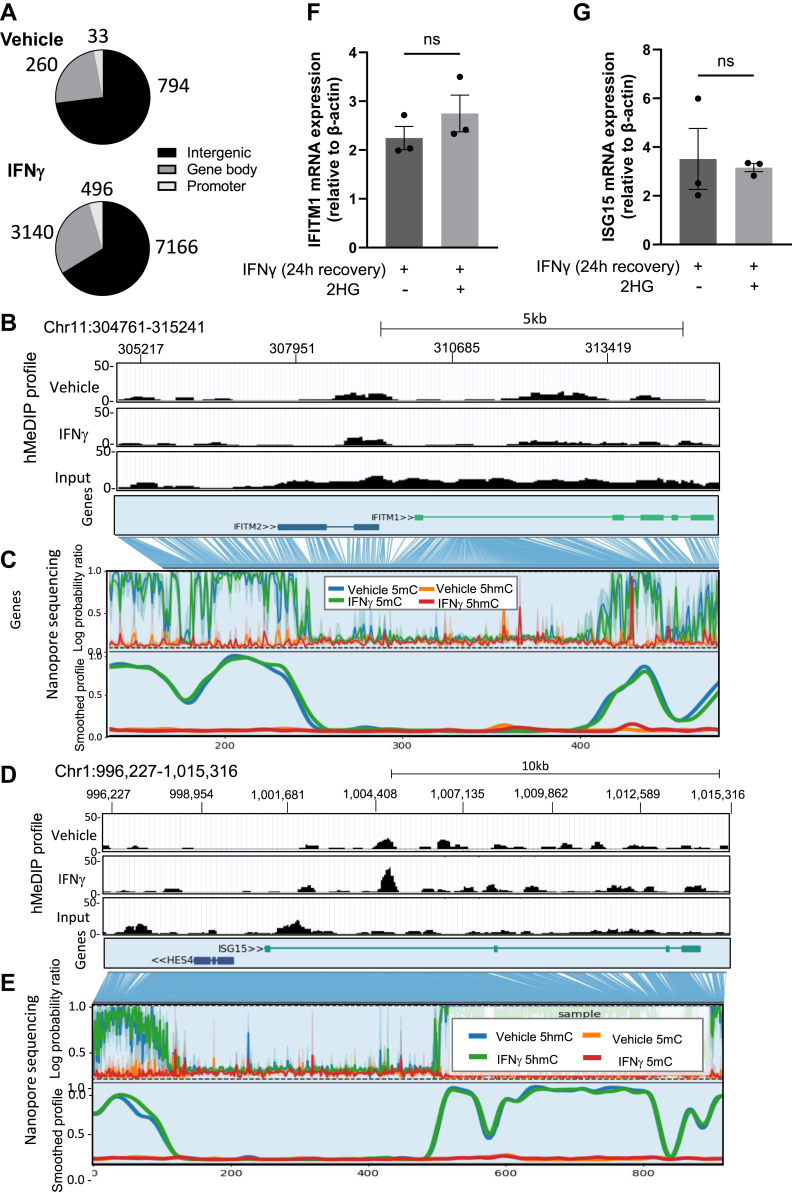
**No change in DNA hydroxymethylation or DNA methylation at IFN-responsive gene loci after IFNγ treatment of HUVEC.** Genomic DNA was prepared from vehicle-treated HUVEC and HUVEC treated for 24 h with 10 ng/ml IFNγ. *A*, hydroxymethylated DNA immunoprecipitation was performed, and the resulting DNA fragments were sequenced alongside input. Pie charts showing the number of 5-hydroxymethylcytosine (5hmC) enrichment peaks in vehicle-treated and IFNγ-treated HUVEC (against input) present in intergenic, gene body, and promoter regions. *B*, raw signal of 5hmC enrichment assessed by hMeDIP-seq at the IFITM1 gene locus. *C*, log probability ratios and smoothed profiles of methylation and hydroxymethylation scores from nanopore sequencing at the IFITM1 gene locus. *D*, raw signal of 5hmC enrichment assessed by hMeDIP-seq at the ISG15 gene locus. *E*, log probability ratios and smoothed profiles of methylation and hydroxymethylation scores from nanopore sequencing at the ISG15 gene locus. 5hmC signal profiles from hMeDIP-seq were visualized using the UCSC genome browser. Nanopore sequencing was visualized using MethylArtist. Chromosome locations refer to the human genome assembly hg38. Coordinates across the Y-axis refer to methylation bins used in the smoothed methylation profile plot. *F-G*, IFITM1 (*F*) and ISG15 (*G*) mRNA expression relative to β-actin were determined by qPCR in HUVEC treated with 10 ng/ml IFNγ for 24 h, followed by its removal for 24 h, all in the presence or absence of 2-hydroxyglutarate (50 μM each L- and D-2HG). A Shapiro-Wilk test for normality was performed, followed by an unpaired t test. Data presented as mean ± SEM. n = 3 triplicate samples.

The catalytic activity of TET family enzymes functions to convert 5mC to 5hmC and subsequent oxidized forms, a crucial step in DNA demethylation. A loss of 5mC and a concomitant increase in 5hmC deposition within promoter or enhancer regions has often been demonstrated (or assumed) to be associated with positive regulation of gene expression. Thus, it might seem surprising that TET2 silencing is here observed to *enhance* gene expression during the IFNγ response. Within gene bodies, however, the abundance of 5hmC has previously been both positively and negatively correlated with gene expression in a cell-type-specific manner (40, 41, 42). To identify changes in the deposition of 5hmC across the genome that occur in ECs upon stimulation with IFNγ, we performed hydroxymethylated DNA immunoprecipitation sequencing (hMeDIPseq). The total number of 5hmC peaks was approximately 10-fold greater in IFNγ-treated HUVEC (10802 peaks) than vehicle-treated controls (1087 peaks), with a greater proportion of peaks being located within gene bodies and promoter regions (Fig. 3*A*) (data are publicly available *via* the GEO database, deposited under GSE232280). We further assessed the promoter region and gene body of IFITM1 for any evidence of altered 5hmC levels following IFNγ treatment that could indicate a role for TET-mediated demethylation in regulating its expression level. The enrichment of 5hmC across the gene body and upstream promoter region was similar between vehicle- and IFNγ-treated HUVEC (Fig. 3*B*). We sought to confirm this finding using a second method and also assess whether levels of 5mC were altered. Using nanopore sequencing, which provides direct, single-base resolution of 5mC and 5hmC presence or absence, we again found no evidence of altered DNA hydroxymethylation or DNA methylation across this locus in control, compared to IFNγ-treated cells (Fig. 3*C*). Similarly, for ISG15, there were no sites at which 5hmC was consistently altered when assessed by both methods, nor was there evidence of loss of 5mC upon IFNγ addition (Fig. 3, *D* and *E*). Consistent with these findings, the levels of IFITM1 and ISG15 upon the administration of IFNγ and subsequent incubation were not affected by the inclusion of 2-hydroxyglutarate, a competitive inhibitor of the catalytic activity of TET enzymes (including TET2) (43) (Fig. 3, *F* and *G*). This led us to conclude that the IFNγ-induced upregulation of classical IFN-responsive genes IFITM1 and ISG15 in ECs is not directly regulated by changes in DNA methylation. Thus, the observed role of TET2 in the regulation of this IFNγ-dependent transcriptional response may be indirect or due to non-catalytic functions.

### TET2 regulates IFNγ-induced expression and release of CXCR3 ligands CXCL9, CXCL10, and CXCL11 in HUVEC

In addition to coordinating the induction of genes involved in anti-viral responses within the infected cell, IFN stimulation leads to upregulation of multiple secreted cytokines in both immune cells and ECs to regulate inflammatory responses. Some cytokines can function to resolve inflammation, whilst others amplify the inflammatory response by recruiting leukocytes to the affected area or activating them once present (44).

**Figure 4:**
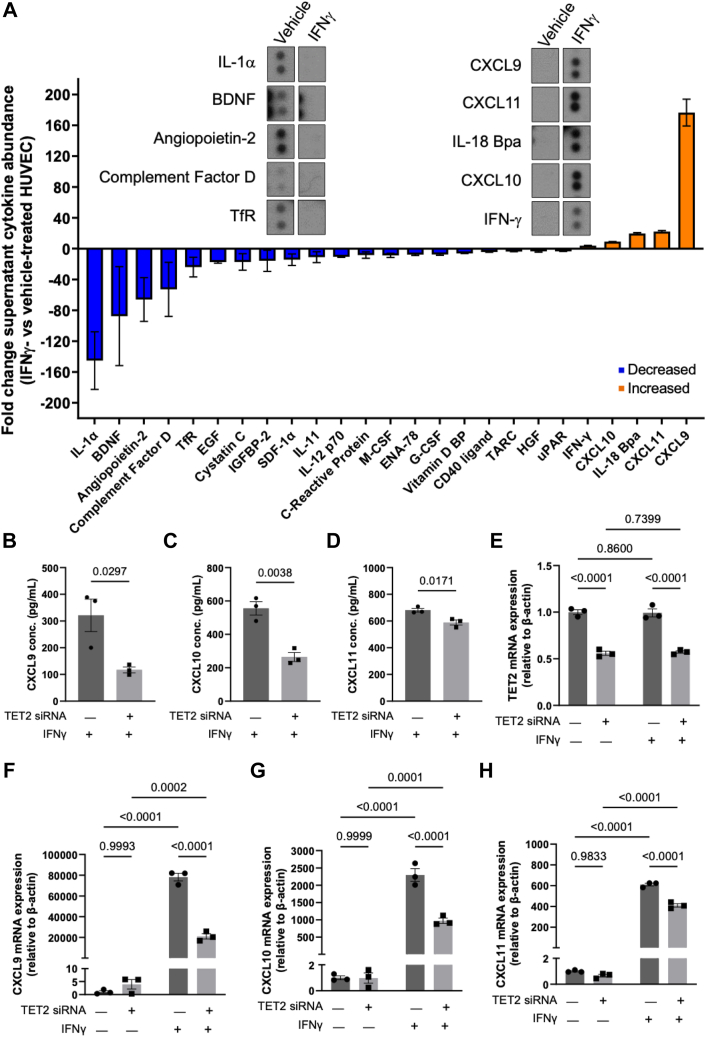
**IFNγ-induced expression of CXCL9, CXCL10 and CXCL11 is decreased by TET2 silencing in HUVEC.***A*, cytokines with >3-fold decreased (*blue*) or increased (*orange*) abundance in the supernatant of 10 ng/ml IFNγ-treated HUVEC compared to vehicle-treated HUVEC after 24 h. Images show the signal of the five cytokines with the greatest increase or decrease in abundance in vehicle- and IFNγ-treated HUVEC. *B-D*, concentration of CXCL9 (*B*), CXCL10 (*C*) and CXCL11 (*D*) present in the supernatant of control and TET2 siRNA-treated HUVEC treated with IFNγ for 24 h, measured by ELISA. A Shapiro-Wilk test for normality was performed, followed by an unpaired *t* test. Data presented as mean ± SEM. n = 3 triplicate samples, representative of three independent experiments. *E-H*, relative mRNA expression of TET2 (*E*), CXCL9 (*F*), CXCL10 (*G*), and CXCL11 (*H*) in siRNA control-treated and TET2-siRNA-treated HUVEC before and after IFNγ treatment for 24 h, measured by RT-qPCR. A 2-way ANOVA and uncorrected Fisher’s LSD with a single pooled variance was performed. Data presented as mean ± SEM. n = 3 triplicate samples, representative of three independent experiments.

To characterize the cytokine profile released from ECs following IFNγ stimulation, the supernatant of HUVEC treated with or without IFNγ for 24 h was collected, and a cytokine array was performed. As would be expected, the different cytokines displayed a diversity of changes in expression as a result of IFNγ treatment (Fig. 4*A*) (See Fig. S5*A* for full cytokine array membrane and Table S1 for full list of fold-changes of identified cytokines). Notably, those with the highest increase in abundance in the supernatant of IFNγ-treated HUVEC included the C-X-C motif-containing chemokines CXCL9, CXCL10, and CXCL11. These are all ligands for CXCR3 (45), and the IFNγ-CXCR3 ligand axis is recognized as a key driver of inflammation in cardiovascular disease and, in particular, in pathologies associated with diabetes (46, 47, 48).

We next determined whether TET2 regulates the IFNγ-dependent activation of these chemokines in HUVEC. Strikingly, the abundance of CXCL9, CXCL10 and CXCL11 proteins were significantly decreased in the supernatant of TET2-silenced HUVEC following IFNγ treatment compared to that of IFNγ-treated control HUVEC (Fig. 4, *B*–*D*). The expression of TET2 itself did not change as a result of IFNγ treatment (Fig. 4*E*). In accordance with the levels of the secreted cytokines (Fig. 4*A*), the mRNA expression levels of CXCL9, CXCL10 and CXCL11 were virtually undetectable at baseline, with no significant difference observed between TET2-silenced HUVEC and controls (Fig. 4, *F*–*H*). The IFNγ-induced mRNA expression of the genes encoding these chemokines increased to a significantly lesser extent in TET2-silenced HUVEC (Fig. 4, *F*–*H*). The extent to which TET2 prevented IFNγ-induced upregulation of the three cytokines at the mRNA level was not equivalent, with the largest magnitude of change observed in CXCL9 expression, followed by CXCL10 and the smallest change detected in CXCL11 expression (Fig. 4, *F*–*H*). The suppressive effect of TET2 depletion upon the IFNγ-induced expression of these cytokines is thus in marked contrast to the *enhancement* of expression of IFITM1 and ISG15.

### Reciprocal changes in methylation and hydroxymethylation at a genomic locus associated with enhancer activity towards CXCL9, CXCL10, and CXCL11 upon IFNγ treatment

**Figure 5:**
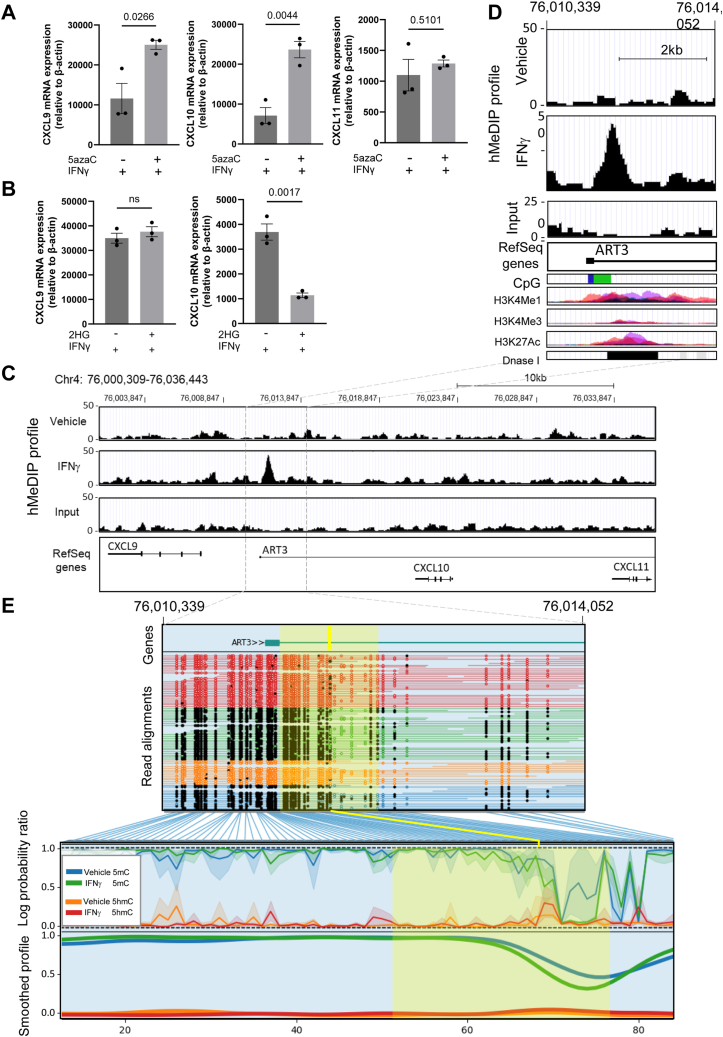
**5hmC enrichment at a regulatory element between CXCL9 and CXCL10 in HUVEC following IFNγ treatment.***A*, CXCL9, CXCL10, and CXCL11 mRNA expression relative to β-actin was determined by qPCR in 10 ng/ml IFNγ-treated HUVEC in the presence or absence of 5-aza-2′deoxycytidine (5azaC), an inhibitor of DNA methyltransferase activity. *B*, CXCL9 and CXCL10 mRNA expression relative to β-actin was determined by qPCR in HUVEC treated with 10 ng/ml IFNγ for 24 h in the presence or absence of 2-hydroxyglutarate (50 μM each L- and D-2HG). *C*, the raw signal profiles from hMeDIPseq of vehicle-treated and IFNγ-treated HUVEC were visualized using the UCSC genome browser to assess 5hmC enrichment at the locus spanning CXCL9, CXCL10, and CXCL11 genes. RefSeq genes are displayed. *D*, inset of region of interest showing peak of 5hmC enrichment and annotations of Weizmann Evolutionary CpG Islands, ENCODE database layered H3K4Me1, H3K4Me3, H3K27Ac, and Dnase I hypersensitivity regions. *E*, nanopore sequencing read alignments showing CpG hydroxymethylation/methylation motifs marked as open or closed dots, log probability ratios, and smoothed profiles of methylation and hydroxymethylation scores visualized using MethylArtist. Chromosome locations refer to the human genome assembly hg38. Coordinates across the Y-axis refer to methylation bins used in the smoothed methylation profile plot. The yellow shaded region marks the location of 5hmC enrichment observed by hMeDIPseq, and the yellow line denotes its peak, mapped to the nanosequencing profile.

We next sought to investigate whether TET2-mediated regulation of CXCL9, CXCL10, and CXCL11 expression in ECs may involve the catalytic activity of TET2, as has been reported in other cells (49). We first tested whether the pharmacological inhibitor of DNA methyltransferases, 5-aza-2′-deoxycytidine (5azaC), would promote the IFNγ-induced expression of the chemokines in HUVEC, and indeed, this was the case for CXCL9 and CXCL10 (Fig. 5*A*), consistent with a loss of DNA demethylation playing a role in the activation of their transcription.

CXCL9, CXCL10, and CXCL11 are located adjacent to one another within a 36 kb region of chromosome 4 (Fig. 5*C*). Notably, a striking increase in 5hmC-enriched DNA was observed by hMeDIPseq in IFNγ-treated HUVEC compared to vehicle-treated controls in a region between CXCL9 and CXCL10 (Fig. 5, *C* and *D*). The region of 5hmC enrichment corresponds to a CpG island and overlaps with H3K4Me1, H3K4Me3, and H3K27Ac marks and a DNAse I hypersensitivity region annotated in the ENCODE database, all of which are often found in regulatory elements (Fig. 5*D*). This locus also comprises the ADP-ribosyltransferase 3 (ART3) gene. Crucially, previous reports have demonstrated the importance of epigenetic marks within this region in the regulation of CXCL9, CXCL10, and CXCL11 expression, suggesting that it acts as an enhancer (50, 51, 52). Using nanopore sequencing, we confirmed the presence of a slightly elevated 5hmC level in IFNγ-treated HUVEC mapping to the same locus (Fig. 5*E*), which also displayed a decreased abundance of 5mC in IFNγ-treated ECs, providing strong evidence that this region is subject to active TET-mediated demethylation by conversion of 5mC to 5hmC (Fig. 5*E*). Furthermore, by contrast to ISG15 and IFITM1, the IFN-γ induction of CXCL10 was highly significantly reduced in the presence of 2-HG (Fig. 5*B*). Perhaps surprisingly, however, the expression of CXCL9 was *unaffected* by 2-HG (Fig. 5*B*; see discussion). Taken together, the identification of a differentially hydroxymethylated region with known regulatory activity toward CXCL9, CXCL10, and CXCL11 upon IFNγ treatment (Fig. 5), combined with our observation that TET2 silencing attenuates their IFNγ-induced expression (Fig. 4), and CXCL10 expression is significantly affected by inhibition of TET activity is indicative that the coordinated upregulation of these chemokines upon IFNγ stimulation, is likely, at least in part, to involve TET2-mediated DNA demethylation at this locus.

### Effects of glucose on TET2-regulated transcription *in vitro*

**Figure 6:**
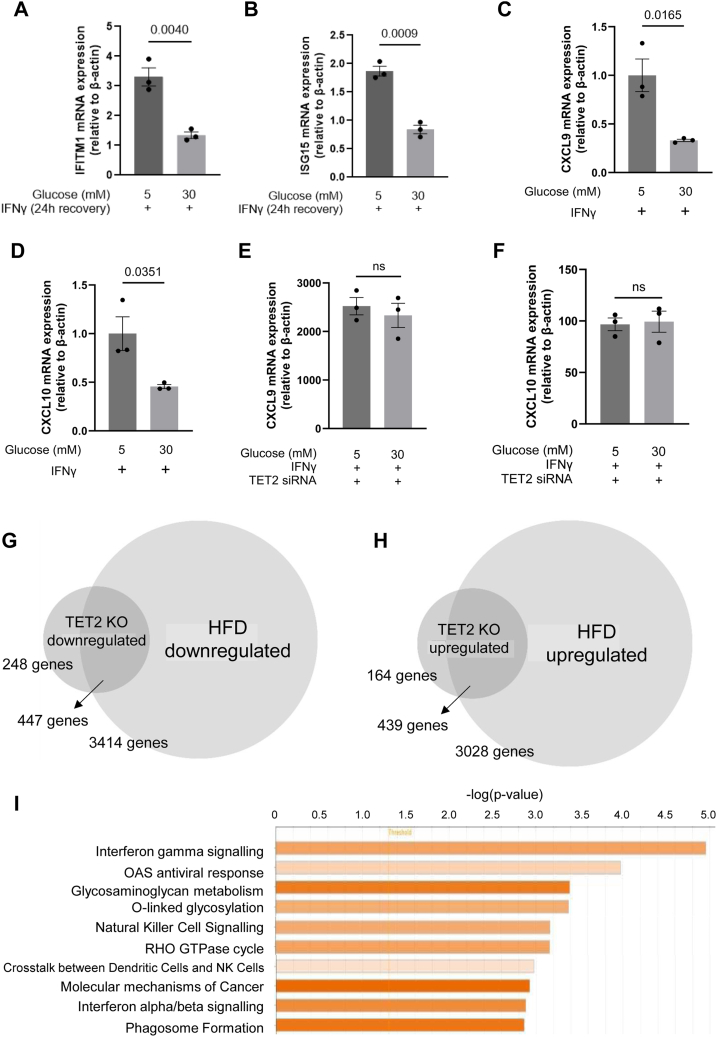
**Overlap in transcriptional dysregulation by TET2 ablation or hyperglycaemia.***A-B*, IFITM1 (*A*) and ISG15 (*B*) mRNA expression relative to β-actin were determined by qPCR in HUVEC cultured for 1 week in high-glucose (30 mM) or normal-glucose conditions (5 mM) with the addition of 25 mM mannitol as an osmotic control after treatment with 10 ng/ml IFNγ for 24 h followed by its removal for 24 h. *C-D*, relative mRNA expression of CXCL9 (*C*) and CXCL10 (*D*) in HUVEC cultured for 1 week under high-glucose conditions (30 mM) or normal-glucose conditions (5 mM) with the addition of 25 mM mannitol as an osmotic control, following IFNγ treatment (10 ng/ml) for the final 24 h. *E-F*, CXCL9 (*E*) and CXCL10 (*F*) mRNA expression relative to β-actin were determined by qPCR in TET2-silenced HUVEC treated with 10 ng/ml IFNγ for 24 h in the presence or absence of high glucose (30 mM) or normal glucose conditions (5 mM) with the addition of 25 mM mannitol as an osmotic control. *G*, overlap between downregulated genes in CD31+ cells from TET2 KO mice (compared to WT mice) or diabetic mice induced by HFD consumption (compared to chow-fed mice). *H*, overlap between upregulated genes in CD31+ cells from TET2 KO mice (compared to WT mice) or diabetic mice induced by HFD consumption (compared to chow-fed mice). Differentially expressed genes (DEGs) were defined as genes with *p* < 0.05. I) Pathway analysis was performed on overlapping DEGs from (*C*) and (*D*). The top 10 pathways predicted to be activated are displayed in order of −log (*p*-value) calculated by Fisher’s Exact Test. Darker orange bars represent higher z-scores.

Decreased expression levels of TET2 together with reduced levels of 5hmC, resulting from high glucose exposure associated with diabetes, have been reported both *in vivo* and *in vitro* (29). However, there remains a paucity of information about the specific genes that are directly (or indirectly) regulated by TET2, which may consequently be dysregulated upon high glucose exposure. A major aim of this study was to determine whether, and to what extent, the transcriptional dysregulation of ECs that occurs in diabetes (which leads to endothelial dysfunction and vascular disease) may be attributable to reduced TET2 activity. The loss of TET2 activity, specifically in HUVEC exposed to high glucose levels *in vitro*, has previously been demonstrated (29). We therefore tested whether high glucose treatment of HUVEC impacted the IFNγ-induced expression assessed here. In the case of IFITM1 and ISG15, glucose exposure significantly blunted the levels of IFNγ-induced expression in both cases (Fig. 6, *A* and *B*). Thus, although this is consistent with glucose-dependent dysregulation of IFN signaling, it may *not* seem consistent with glucose acting solely to inhibit TET2 activity to elicit this effect (since TET2 silencing acted to increase expression of these genes). However, high glucose treatment also significantly blunted the activation of expression of CXCL9 and CXCL10 in response to IFNγ (Fig. 6, *C* and *D*). Furthermore, the effect of high glucose was, in these cases, ablated in the absence of TET2 (Fig. 6, *E* and *F*). These data *would* therefore suggest that, in the case of the IFN-dependent transcriptional regulation of CXCL9 and 10, TET2 is a positive regulator that can be inhibited by high glucose exposure.

### Endothelial IFN signaling and glycosaminoglycan biosynthesis are commonly dysregulated by both TET2 depletion and a diabetic model

Next, turning to an *in vivo* approach, we used a model of type 2 diabetes induced by high-fat diet (HFD) feeding (confirmed to display impaired glucose tolerance and significantly elevated fasting blood glucose levels, as shown in Fig. S4, *B*–*D*) and characterized the transcriptional changes that occurred in CD31+ve lung microvascular cells. We compared these changes to those that resulted from EC-specific TET2 ablation in mice (Fig. 6, *G* and *H*). In this analysis, we broadened our criteria (from those applied to Fig. 2) to include all genes with *p* < 0.05 with no fold-change cut-off, since for some genes, even a small alteration of transcript number may have profound biological effects, and that any similarity in alteration of transcript abundance by TET2 ablation and hyperglycemia could indicate a common mode of regulation. HFD consumption induces a plethora of phenotypic changes such as obesity and insulin-resistance as well as hyperglycemia and thus, as expected, the transcriptome of microvascular ECs from these mice was substantially altered, with 3861 genes downregulated and 3467 genes upregulated compared to standard chow-fed mice signaling, representing about 10% of all genes detected in this screen, in each case. Endothelial TET2 ablation resulted in upregulation of 603 genes and downregulation of 695 genes using the cut-off described above. Strikingly, of the genes dysregulated by TET2 ablation, the overwhelming majority – almost 70%- were differentially expressed in the same direction as in the diabetic model (Fig. 6, *G* and *H*). We further subjected the overlapping lists of differentially expressed genes to Ingenuity Pathway analyses and, as might be expected from the high degree of overlap, identified similar pathways to those resulting from TET2 depletion (Fig. 6*I*). In particular, these analyses reflected highly significant, upregulated IFN-related signaling pathways in both the TET2 KO and diabetic cases. Further, glycosaminoglycan (GAG) metabolism emerged as an important biological function that was similarly impacted in both the TET2KO and diabetic mouse models. GAGs are critical components of the endothelial glycocalyx, and their dysregulation in hyperglycemia is well recognized to contribute to endothelial and vascular dysfunction in diabetes (53). A list of the specific dysregulated GAG-associated genes and their potential roles in endothelial (dys)function and/or diabetes-associated vascular disease is given in Table 1 (54, 55, 56, 57, 58, 59, 60, 61, 62, 63, 64).

**Table 1:** Table of genes involved in glycosaminoglycan metabolic pathways that are similarly impacted in ECs by TET2 depletion and high fat diet-induced diabetes

Gene	Role in cardiovascular disease in diabetes and/or endothelial (dys)function	Reference
UDP-GlcNAC:betaGal beta-1,3-N-acetylglucosaminyltransferase 3 (B3GNT3)	Contributes to glycosylation during the advancement of diabetes.	(54)
carbohydrate sulfotransferase 1 (CHST1)	Upregulation enhances leukocyte interactions with endothelium at sites of inflammation.	(55)
carbohydrate sulfotransferase 2 (CHST2)	Upregulation enhances leukocyte interactions with endothelium at sites of inflammation.	(55)
heparan-alpha-glucosaminide N-acetyltransferase (HGSNAT)	Mutations lead to inherited metabolic disorders linked to cardiovascular disease.	(56)
hyaluronidase 1 (HYAL1)	Dysregulated (over)expression contributes to endothelial and glycocalyx dysfunction induced by diabetes.	(57)
hyaluronidase 2 (HYAL2)	Implicated in glycocalyx impairment under low shear stress.	(58)
N-deacetylase and N-sulfotransferase 1 (NDST1)	Contributes to interstitial inflammation under diabetic conditions.	(59)
N-sulfoglucosamine sulfohydrolase (SGSH)	Catalyzes the lysosomal degradation of heparan sulfate. Misexpressed in magnesium-deficient, dysfunctional ECs.	(60, 61)
ST3 beta-galactoside alpha-2,3-sialyltransferase 1 (ST3GAL1)	Upregulated in human retinal microvascular ECs in hyperglycemia.	(62)
ST3 beta-galactoside alpha-2,3-sialyltransferase 2 (ST3GAL2)	Transcriptionally regulated by hypoxia in pulmonary endothelium suggests a role in functional adaptation to oxygen levels.	(63)
ST3 beta-galactoside alpha-2,3-sialyltransferase 4 (ST3GAL4)	Upregulated in human retinal microvascular ECs in hyperglycemia.	(62)
ST3 beta-galactoside alpha-2,3-sialyltransferase 6 (ST3GAL6)	Promotes neutrophil recruitment during inflammation.	(64)

## Discussion

There is mounting evidence that TET2 plays an important functional role in the vascular system, with previous studies identifying roles for TET2 in regulating the phenotypic plasticity of vascular smooth muscle cells (27) and regulating autophagy and the production of vasoactive substances by ECs (25, 28). Studies have demonstrated the importance of TET2 in vascular disease, as downregulation of TET2, and correspondingly, decreased abundance of 5hmC (the first product of TET catalytic activity), are associated with the severity of atherosclerosis and vascular injury in humans and murine models (25, 26, 27). However, the consequences of the loss of TET2 specifically in the endothelium *in vivo* have not, thus far, been reported, and the specific targets of TET2-mediated gene regulation in ECs have not been explored in an unbiased fashion.

In this study, we assessed the role of endothelial-expressed TET2 in the control of vascular tone using *ex vivo* tension measurements of aortic rings since previous *in vitro* studies have identified TET2-dependent regulation of eNOS, CSE, and endothelin-1 (25, 28). However, we observed no difference in the responses of aortic rings to PE or ACh between WT and TET2 KO mice under baseline conditions. It is possible that TET2 might contribute to the regulation of vascular tone in vascular beds other than the aorta. However, in our other studies, we have not found evidence of altered blood pressure resulting from endothelial-specific depletion of TET2 (under baseline conditions; unpublished observations), as might be expected to result from the altered expression of vasoactive substances, particularly within ECs of resistance vessels (65). It is, however, noteworthy that the TET2-mediated regulation of both eNOS and endothelin was observed under conditions of low shear stress, while regulation of the CSE/H_2_S system was observed in the setting of elevated oxidized LDL (25, 28). Future studies might therefore assess the involvement of endothelial TET2 in regulating vascular tone under pathological, stressed conditions, such as in atherosclerosis-prone mice.

We performed an unbiased transcriptome analysis of TET2 depleted, compared to wild-type, ECs *in vivo*. A striking finding of our study was the observation that TET2 acts as a negative regulator of IFN-response genes in ECs *in vivo*. Thus, the response to IFN, and most noticeably IFN-γ, was identified as a significantly upregulated pathway in TET2 KO mice (Fig. 2*C*). Notably, this transcriptional signature was also evident in the ECs of diabetic mice. Reports from studies in multiple cell types provide evidence that high glucose exposure perturbs the expression of genes involved in IFN signaling pathways (66, 67, 68, 69, 70, 71). Our data further support these findings and demonstrate, at least in the case of the IFNγ-induced expression of CXCL9 and CXCL10 cytokines, that the dysregulation by glucose can be attributed to inhibition of TET2. Furthermore, there are reports that differential methylation of IFN response genes under high glucose conditions correlates with their altered expression in particular cell types and in blood samples of diabetic individuals (66, 67, 68, 69). Taken together therefore, these data strongly support the hypothesis that the vascular disease which is prevalent in diabetics is in part due to compromised TET2 expression and/or activity within the endothelium and that this involves dysregulated IFN responses. (29). The metabolism of GAGs was also identified in our screens as commonly dysregulated both by TET2 depletion and in a diabetic model. As principal components of the glycocalyx of the vascular endothelium, the consequences of their dysregulation during diabetes upon vascular dysfunction is well recognized (53). It has also been shown in several studies in fibroblasts that GAG metabolism is impacted by IFNγ signaling (72, 73, 74, 75, 76). Whether the dysfunction of the glycocalyx in diabetes-associated vascular disease is, in part, driven by altered IFNγ signaling, consequent by TET2 depletion therefore warrants future investigation (77).

We confirmed in HUVECs, *in vitro*, that TET2 acts as a negative regulator of the well-characterized IFN-response genes, ISG15 and IFITM1, upon IFNγ exposure. Notably, the increase in expression, upon TET2 depletion, was most evident after removal of the IFNγ, as the expression of the genes continued to rise significantly, by marked contrast to the expression profile in the control cells (Fig. 2*E*). This might suggest the involvement of TET2 in the resolution phase of the immune response. Excessive or prolonged IFNγ signaling is associated with chronic inflammation and development of atherosclerosis, including in diabetes (38), and so the potential role of the loss of TET2 in this process merits further exploration. Mechanistically, however, we have not found evidence for the direct involvement of catalytic TET activity or changes in DNA methylation associated with IFNγ-mediated stimulation of transcription within the gene loci of ISG15 or IFITM1. Whether TET2 acts to suppress their expression *via* a non-catalytic mechanism or perhaps catalytic activity towards upstream regulators warrants further study. Given that the stability of TET2 protein is affected by high glucose levels, impeding its AMPK-mediated phosphorylation and leading to calpain-mediated degradation (29), high glucose levels may likely impact its non-catalytic as well as catalytic roles. One previously reported non-catalytic role of TET2 is its requisite physical interaction with O-GlcNAc transferase (OGT) to regulate the histone H3K3 methyltransferase SET1/COMPASS (78). Increased production of UDP-GlcNAc (which is transferred by OGT to proteins for glycosylation) is likely in hyperglycemia due to increased flux through the hexosamine biosynthesis pathway, providing another plausible mechanism by which glucose levels may disrupt non-catalytic activities of TET2 in gene regulation (79, 80).

We further demonstrated in this study that TET2 is a *positive* regulator of the IFNγ-induced expression of the CXCR3 ligands, CXCL9, CXCL10, and CXCL11, in ECs. These were found to be amongst the most abundant cytokines released by HUVEC upon IFNγ treatment and, by contrast to other classical IFN-responsive genes tested, their upregulation during IFNγ treatment was significantly blunted by TET2 silencing. The involvement of TET2 in the upregulation of these cytokines has been demonstrated previously in B16-OVA melanoma cells (49). Further, in that study, the direct, IFNγ-dependent binding of TET2 to the CXCL10 promoter was demonstrated, concomitant with enriched levels of 5hmC, consistent with a direct role for DNA demethylation in the transcriptional activation of the cytokines. Our study similarly identified, by hMeDIP-sequencing, a region between the CXCL9 and CXCL10 gene loci, in which 5hmC abundance was increased upon IFNγ treatment, again consistent with the direct involvement of changes in DNA methylation in the observed transcriptional activation. This was confirmed independently by nanopore sequencing, which showed a small gain of 5hmC corresponding to a loss of 5mC at this region. Such a relationship between 5hmC and 5mC is typically assumed but has often proved challenging to demonstrate experimentally. Our observations provide further evidence that this region within the CXCL9/10/11 gene locus may act as an enhancer, and provide the novel insight that altered (hydroxy)methylation of the region is likely to be involved in gene regulation, in addition to the histone modifications previously reported (50, 51, 52).

We have here only demonstrated the function of TET2 in the activation of the transcription of the CXCL9/10/11 cytokines by IFNγ. Our data from qPCR experiments indicate TET2 transcripts are more abundant than either TET1 or TET3 in HUVECs (Fig. S5*C*). However, data mined from 128 publicly accessible RNA sequencing databases (accessed *via* BulkECexplorer (81)), demonstrate all 3 TETs to be expressed in a range of ECs and thus it cannot be completely ruled out that TET1 and/or TET3 might also contribute to the observed changes in methylation (Fig. S5, *D*–*F*). Unique to the three TET family members, TET2 does not possess a CXXC DNA-binding domain (82). TET2 is therefore understood to partner with other proteins to facilitate its interaction with DNA. A variety of binding partners including transcription factors have been identified to recruit TET2 and activate expression of target genes (29, 49, 78, 82, 83, 84, 85, 86, 87, 88, 89, 90, 91, 92). Notably, in THP-1 and B16-OVA cells, an interaction between STAT1, a transcription factor known to mediate IFN signaling *via* the IFN/JAK/STAT pathway (93), and TET2 was identified, which could be increased by IFNγ (49). Both CXCL9 and CXCL10 contain STAT1 binding elements within ∼200 bp upstream of their transcriptional start sites (94), and it is possible that STAT1 recruits TET2 to mediate hydroxymethylation and regulate this gene expression in ECs. However, this remains to be tested experimentally, and it should be noted that these binding sites do not correspond to the region at which we observed differential hydroxymethylation upon IFNγ treatment.

Functionally, CXCL9, CXCL10, and CXCL11 are involved in the chemoattraction of leukocytes, particularly CD8+ and CD4+ T cell populations, which abundantly express the CXCR3 receptor for which these cytokines are ligands (95). They are known to have critical protective roles in the immune system, recruiting activated T cells to sites of infection and consequent enhancement of anti-tumor immunity, together with restraint of tumor growth (reviewed in (96)). However, studies have indicated that CXCL11 invokes an alternative signaling cascade to CXCL9 and CXCL10 (97, 98). Thus, whereas CXCL9 and CXCL10 have predominantly pro-inflammatory effects downstream of ligand binding (promoting Th1/Th17 cell development), CXCL11 instead signals to recruit the Tr1 subtype and promotes CXCR3 receptor internalization, thereby promoting the resolution of inflammation. Although in our study the expression of all three cytokines was reduced significantly upon TET2 depletion, the change in expression of CXCL11 was much smaller in magnitude than that of the other two cytokines. In addition, CXCL11 expression was not significantly changed by 5-azaC treatment. Given that the regulatory region identified lies in closer proximity to the CXCL9 and CXCL10 genes, the expression of CXCL11, which is more distant from the regulatory region, may be less affected by changes in the methylation status of the identified locus and account for these differences. Differential regulation of these cytokines by DNA methylation could plausibly be of biological importance in coordinating the timing of pro- and anti-inflammatory signals. The observation here that although both CXCL9 and CXCL10 were significantly positively regulated by TET2 in the context of IFN-signalling, yet only CXCL10 expression was found to be dependent upon catalytic TET activity, potentially adds another layer of complexity to the coordinated regulation of expression of these cytokines.

*In vivo*, we did not observe significant changes in the mRNA transcripts of CXCL9/10/11 in the ECs of either our TET2KO or diabetic mouse models. Neither were there any significant changes in the serum levels of these cytokines apparent in these mice (Fig. S5*B*). This is likely due to the fact that CXCR3 ligands have predominantly been ascribed antimicrobial activity (99), and the conditions under which the mice are housed preclude their exposure to microbes and significant immune upregulation. However, altered levels of the cytokines have been frequently observed in the plasma of diabetics (reviewed in (100, 101)), consistent with their dysregulation in this clinical setting. While the majority of studies report increased levels (which might not seem consistent with our findings here) a meta-analysis identified a significant *decrease* of plasma CXCL10 associated with patients with gestational diabetes (102). Further, expression of the cytokines can be induced in many other cells, most notably macrophages (103), contributing to plasma levels. The concentration of chemokines localised to a specific site of inflammation or infection may have a greater dependence on endothelium-derived chemokines than whole plasma. Thus, the potential effects of TET2 depletion (as a result of hyperglycemia) specifically in the endothelium of diabetics warrant further investigation.

In conclusion, our study has identified endothelial-expressed TET2 to be an important regulator of IFNγ transcriptional responses, likely acting through diverse (both catalytic and non-catalytic) mechanisms, and affecting both activation and resolution of immune responses. A striking similarity in the EC transcriptome of TET2KO mice, compared to diabetic mice, is consistent with previous observations that hyperglycemia acts to suppress the levels and catalytic activity of TET2. In particular, our data might suggest that perturbation of TET2 activity in the endothelium, as a consequence of hyperglycemia may contribute to dysfunctional activation and resolution of IFNγ responses in diabetes. This, in turn, could account for the increased susceptibility of diabetics to recurrent viral infections (104, 105), and the chronic inflammatory state, which contributes to the long-term diabetic vascular complications (106).

A limitation of our study is that the high-fat diet mouse model can impact upon endothelial homeostasis by mechanisms not directly involving hyperglycemia, such as dyslipidemia and hyperinsulinemia. However, as this model was in use concurrently for other studies, its use here is justified by the ethical principle in the UK to observe the 3Rs (reduction, refinement, replacement of animals in research). Furthermore, our study includes only one sex of mice. Male mice were chosen for this study based on previous literature demonstrating a greater effect of high-fat diet feeding on glucose tolerance in male C57BL6/J mice compared to female mice (107). It must be acknowledged that these findings may differ in female mice. Nonetheless, our *in vivo* and *in vitro* data are consistent with a (post-translational) loss of TET2 activity, impacted by high glucose exposure, that contributes to hyperglycemia-induced endothelial dysfunction *via* the dysregulated transcriptional regulation of endothelial IFN responses. Therefore, further research into the potential of TET2 as a therapeutic target for limiting endothelial dysfunction in hyperglycemia-induced vascular disease is warranted. Such research should consider the study of multiple vascular beds and sub-populations of ECs, as substantial heterogeneity across tissue and vessel types has been observed in recent single-cell transcriptomic analyses (108, 109).

## Experimental procedures

### Animal studies

All studies were conducted in accordance with the Guidance on the Operation of the Animals (Scientific Procedures) Act, 1986 (UK Home Office) and with ethical approval from King’s College London Animal Welfare Ethical Review Body. Mice were housed in individually ventilated cages in a temperature and humidity-controlled environment with a 12 h light/dark cycle (light phase 7 AM-7 PM) with access to food and water *ad libitum*. Experimental mice were generated by crossing TET2^fl/fl^ mice (30) to tamoxifen-inducible, EC-specific Cre-expressing mice (Cdh5-CreERT2) (31) on a C57Bl/6J background. Male Cre^+ve^ TET2^fl/fl^ mice and male Cre^−ve^ TET2^fl/fl^ mice were injected intraperitoneally with 40 mg/kg tamoxifen (MP Biomedicals, #0215673894) in peanut oil for three consecutive days at 6 to 8 weeks of age to induce Cre recombination or generate appropriate control animals. Body weights were measured at 4-week intervals. Mice appeared behaviorally normal, and no differences in body weight were apparent between genotypes (Figs. S2*A* and S3*A*).

Where indicated, male Cre^-ve^ TET2^fl/fl^ mice were continued on a standard chow diet (13% kcal from fat, LabDiet 5053) or changed to a high-fat diet (60% kcal from fat, TestDiet 58Y1) *ad libitum* for 20 weeks to induce a diabetic phenotype. Glucose tolerance tests were performed at 9 weeks of high-fat diet (or standard chow diet) feeding. Mice were fasted for 4 h, and blood was sampled from the tail vein using the needle-prick method. A baseline blood glucose concentration was measured using a Sinocare Safe-Accu two blood glucose monitor. Following intraperitoneal injection of D-glucose in saline (2 g/kg dose), further blood samples were measured at 15, 30, 45, 60, and 120 min. Increased body weight, impaired glucose tolerance, and increased fasting blood glucose concentration were confirmed in the high-fat diet-fed group (Fig. S4).

### Genotyping

Ear clips were taken from mice after weaning and digested with 300 μl 50 mM NaOH for 30 min at 95 °C. Samples were vortexed before the addition of 50 μl 1 M Tris-HCl and centrifugation at 16,000×g for 5 min 2 μl of the resulting supernatant was mixed with 12.5 μl GoTaq G2 Master Mix (Promega, #M7823), 2 μl of 10 μM primers (GAPDH forward: CCTAGACAAAATGGTGAAGG GAPDH reverse: GACTCCACGACATACTCAGC Cre forward: TGCCAGGATCAGGGTTAAA Cre reverse: CCCGGCAAAACAGGTAGTT),and 8.5 μl H2O. PCR amplification was performed as follows: 94 °C 1 min, 35 cycles (94 °C 30 s, 60 °C 30 s, 72 °C 30 s), 72 °C 7 min, 4 °C. 10 μl PCR products were separated by size on a 1.5% agarose gel containing Nancy 520 (Sigma #01494) for visualization with 1× TAE buffer (Thermo Fisher Scientific, #B49) at 100 V, alongside 5 μl of 100 bp ladder (New England Biolabs, #N0551S) to enable identification of band size. The presence or absence of a 200 bp band was used to genotype mice as Cre^+ve^ or Cre^−ve^. The presence of a GAPDH band at 342 bp was confirmed in all samples as a housekeeping gene.

### Aortic ring organ bath experiments

Male Cre^+ve^ and Cre^−ve^ mice were sacrificed at 20 or 30 weeks of age ( ± 1 week) by intraperitoneal injection of 300 μl of 200 mg/ml sodium pentobarbital, and death was confirmed by cervical dislocation or exsanguination. The aorta was carefully removed, placed in ice cold Krebs buffer (NaCl 6.95 g/L, KCl 0.35 g/L, KH2PO4 0.16 g/L, NaHCO3 2.1 g/L MgSO4.7H2O 0.29 g/L, Glucose 1.98 g/L, CaCl2.2H2O 0.37 g/L) and dissected to clear perivascular fat and surrounding tissues whilst avoiding damage to the endothelium. 3 mm aortic rings were mounted onto wire triangles and suspended in an organ bath containing Kreb’s buffer, maintained at 37 °C and continuously aerated with 95% O2 and 5% CO2. Tension was increased from 0.5 g to 3 g gradually by 0.5 g every 5 min and then allowed to reach a stable baseline. 40 mM KCl was applied to test the constriction response, washed out and repeated once. Aortic rings that did not increase tension by ≥ 10% in response to the second application of KCl were excluded. Following 20 min of acclimatization, aortic rings were pre-constricted by the addition of 3x10-9 M phenylephrine (PE) and then exposed to an increasing dose of acetylcholine (ACh), between 10^−9^ M and 10^−5^ M, applied at 3 min intervals. Aortic rings that failed to relax to >50% of the PE-induced constriction or relaxed by >140% were excluded to rule out vessels that may have been damaged during preparation. After washing and a 20-minute interval, PE-induced constriction was measured in a range of doses increasing from 10^−9^ M to 10^−5^ M. Finally, aortic rings were pre-constricted with 3 × 10^−9^ M PE and a sodium nitroprusside relaxation curve was performed in a dose range between 10^−11^ M and 10^−5^ M.

### Isolation of CD31+ cells from murine lungs

TET2^fl/fl^ Cre^+ve^ and TET2^fl/fl^ Cre^−ve^ male, tamoxifen-injected mice (fed either a standard chow or high fat diet) were sacrificed at 30 weeks of age by intraperitoneal injection of 300 μl of 200 mg/ml sodium pentobarbital and death was confirmed by cervical dislocation. Lungs were removed and washed twice with PBS containing 1% penicillin-streptomycin before finely mincing with scissors. Minced lung tissue was digested in 1 mg/ml collagenase-dispase (Merck, #10269638001) in Dulbecco's Modified Eagle Medium (DMEM) for 45 min at 37 °C with continuous shaking before passing through a 70 μm nylon strainer followed by a 30 μm nylon strainer, washing with PBS to ensure all cells passed through the filters. The cell suspension was centrifuged at 350×*g* for 10 min, the supernatant discarded and the resulting cell pellet was resuspended in 5 ml PBS before a further 5 min centrifugation at 350×*g*. The supernatant was removed and the cell pellet was resuspended in 90 μl of 4 °C MACS buffer (1:20 dilution of MACS BSA stock solution Miltenyi, #130-091-376 in autoMACS rinsing solution Miltenyi, #130-091-222). 10 μl of anti CD31 antibody-conjugated microbeads (Miltenyi, #130-097-418) were added and incubated at 4 °C for 15 min, followed by magnetic separation using a MACS separator and LS columns (Miltenyi #130-042-401). CD31+ cells in suspension were centrifuged at 350×*g* for 5 min and the supernatant was discarded.

### Confirmation of Cre-recombination

CD31+ cells were digested in 150 μl 50 mM NaOH at 95 °C for 30 min 25 μl 1 M Tris-HCl was added and samples were centrifuged at 16,000×*g* for 5 min 2.5 μl of the resulting supernatant was mixed with 12.5 μl GoTaq G2 Master Mix (Promega, #M7823), 2 μl of 10 μM primers (Combinations of Tet2FloxF: AAGAATTGCTACAGGCCTGC; Tet2FloxR: TTCTTTAGCCCTTGCTGAGC; Tet2LoxP3R: TAGAGGGAGGGGGCATAAGT as displayed in Fig. S1) and 8 μl H_2_O. PCR amplification was performed as follows: 94 °C 1 min, 35 cycles (94 °C 30 s, 56 °C 30 s, 72 °C 30 s), 72 °C 7 min, 4 °C. 13 μl PCR products were separated by size on a 1.5% agarose gel (containing Nancy 520 for visualization) with 1× TAE buffer at 120 V, alongside 5 μl of 100 bp ladder to enable identification of band size.

### RNA sequencing of CD31+ cells

RNA was prepared from CD31+ cells immediately after isolation using the Reliaprep kit (Promega, #G9711). 350 ng RNA from n = 3 individual mice per group (30-week old WT and TET2 KO mice) was used for library preparation, sequencing (50 M reads) and initial bioinformatic analysis by Novogene, as follows:

mRNA was purified from total RNA using poly-T oligo-attached magnetic beads. After fragmentation, the first strand cDNA was synthesized using random hexamer primers, followed by the second strand cDNA synthesis. cDNA underwent end repair, A-tailing, adapter ligation, size selection, amplification, and purification. The library was checked with Qubit and real-time PCR for quantification and bioanalyzer for size distribution detection. Library preparations were sequenced on an Illumina platform, and paired-end reads were generated. Raw data were first processed through in-house perl scripts to generate clean reads by removing reads containing adaptors, reads containing poly-N and low-quality reads. Clean reads were aligned to the reference genome using HISAT2 software and gene expression was quantified using featureCounts software. Differential gene expression analysis was performed using the DESeq2 R package. Further analysis was performed using Ingenuity Pathway Analysis (IPA) software (Qiagen). For analyses presented in Figure 2, thresholds of Log2FoldChange>|1| and *p* < 0.05 were applied. Data are publicly available *via* the GEO database, deposited under GSE232888.

### Cell culture

HUVEC from pooled donors (Lonza, #C2519A) were expanded in endothelial growth media-2 (EGM-2 #CC-3156 and #CC-3162) media (Lonza), in T75 flasks coated with 0.4% gelatin (Sigma, #G1393) in phosphate buffered saline (PBS). HUVEC were frozen in heat-inactivated fetal calf serum (FCS) (Sigma, #F9665) with 10% dimethyl sulfoxide (DMSO) and stored in liquid nitrogen at passage 3. HUVEC were thawed and cultured in EGM-2 media (Promocell, #C-22011) supplemented with 1% penicillin-streptomycin in 0.4% gelatin-coated T75 flasks in a 37 °C humidified incubator with 5% CO_2_. All HUVEC experiments were conducted at passage 4.

### siRNA transfection

HUVEC were seeded at approximately 3 × 10^5^ cells per well of a 6-well plate in EGM-2 media. After 24 h, media was replaced with optiMEM (Thermo Fisher Scientific #11058021) for 1 h prior to transfection. SilencerTM Select siRNAs (Thermo Fisher Scientific, #4392420 TET2: s29441 and Negative Control No. 1 #4390843) were mixed with lipofectamineTM RNAiMAX (ThermoFisher Scientific, #13778075) and diluted 1:1 with lipofectamine RNAiMAX (1:100 in optiMEM). Mixtures were incubated at room temperature for 15 min before applying to cells in triplicate, to a final concentration of siRNA 20 nM per well in 800 μl optiMEM containing 8 μl lipofectamine RNAiMAX. Media was replaced with EGM-2 after 7 h. HUVEC were harvested 48 to 72 h after transfection.

### 5-Aza-2′deoxycytidine (5azaC) treatment

Where indicated, HUVEC were treated with 5 μM 5-aza-2′-deoxycytidine (5azaC) (Sigma #A3656) or 0.1% DMSO (as vehicle controls) in EGM-2 for 72 h, refreshing the treatment every 24 h. For the final 24 h, IFNγ was added at 10 ng/ml.

### 2-Hydroxyglutarate treatment

Where indicated, HUVEC were treated with 50 μM each of D-2-hydryoxyglutarate and L-2-hydroxyglutarate (2HG) in EGM-2 for 24 h, in combination with IFNγ at 10 ng/ml. In some experiments, IFNγ-containing media was removed after 24 h and replaced with EGM-2 containing 2HG for a further 24 h.

### High glucose treatment

To simulate hyperglycemia, 25 mM D-glucose was added to EGM-2 media for a final concentration of 30 mM glucose. To control for osmotic effects, control HUVEC in EGM-2 (5 mM glucose) were supplemented with 25 mM D-mannitol. Treatment continued for 1 week, with media replenished every 2 to 3 days. For IFNγ experiments, IFNγ was added at 10 ng/ml for the final 24 h. In some experiments, IFNγ-containing media was removed after 24 h and replaced with EGM-2 containing 30 mM D-glucose or 5 mM glucose with 25 mM D-mannitol for a further 24 h before harvesting. For experiments including TET2 silencing, HUVEC were pre-treated with D-glucose or D-mannitol before siRNA transfection as described above for 7 h, then returned to EGM-2 media with D-glucose or D-mannitol for the remainder of the experiment, with IFNγ added and removed as indicated (48–72 h transfection, total 1-week high glucose/mannitol).

### RT-qPCR (SYBR green)

RNA was extracted using a Reliaprep kit (Promega, #Z6012), according to the manufacturer’s instructions (with the exceptions that DNA was sheared with Qiashredders (Qiagen, #79656) before addition of isopropanol and that 1 μl RQ1 RNase-free DNAse (Promega, #M6101) per sample was included in addition to DNase I in the incubation mix). RNA was eluted in nuclease-free H_2_O and stored at −80 °C. 500 ng RNA was diluted to a volume of 8 μl in nuclease-free H_2_O with the addition of 2 μl 5× LunaScript RT SuperMix (New England Biolabs, #E3010 L). Samples underwent primer annealing at 25 °C for 2 min, followed by cDNA synthesis at 55 °C for 10 min and then heat inactivated at 95 °C for 1 min. Resulting cDNA samples were diluted to a final volume of 100 μl with nuclease-free H_2_O. Negative controls were also prepared using equivalent amounts of RNA, H_2_O and the No-RT Control Mix. Primer pairs were designed to span an intron in the corresponding genomic DNA using the NCBI Primer-BLAST online tool (https://www.ncbi.nlm.nih.gov/tools/primer-blast). qPCR was performed with SYBR green mastermix (PCR biosystems, #PB012612-04), forward and reverse primers (to a final concentration of 300 nM) (Table 2), 2 μl cDNA and nuclease-free H_2_O to a final 20 μl reaction volume using the StepOne Plus Real-Time PCR system (Applied Biosystems). Duplicate samples underwent denaturation for 10 min at 95 °C, followed by amplification by 40 cycles of 95 °C for 15 s followed by 60 °C for 30 s. Melting profiles were also conducted to confirm specificity of primers by the appearance of a single melt curve peak indicating a single amplicon. Relative mRNA expression was calculated using the 2-ΔΔCT method.

**Table 2:** Sequences of primers used for RT-qPCR

Species	Gene	Forward primer sequence (5′-3′)	Reverse primer sequence (5′-3′)
Human	RPLP0	CCATTCTATCATCAACGGGTACAA	TCAGCAAGTGGGAAGGTGTAATC
Human	TET2	GAAAGGAGACCCGACTGCAA	ATCTTGAGAGGGTGTGCTGC
Human	IFITM1	CGGCTCTGTGACAGTCTACC	CTGCTGTATCTAGGGGCAGG
Human	ISG15	ACAGCCATGGGCTGGGA	CCTTCAGCTCTGACACCGAC

### RT-qPCR (Taqman)

For CXCL9, CXCL10 and CXCL11, pre-designed Taqman primers/probes (Thermo Fisher) were used (CXCL9 (#Hs00171065_m), CXCL10 (#Hs00171042_m1), CXCL11 (#Hs00171138_m1), β-actin (#Hs99999903_m1)). Assay conditions were used in accordance with the manufacturer’s protocol and with 1 μl cDNA template. The assay reaction conditions were as follows: Stage 1 (holding stage) 50 °C for 2 min. Stage 2 (polymerase activation) 95 °C for 20 s. Stage 3 (cycling stage – 40 cycles) 95 °C for 1 s, 60 °C for 20 s. All samples were performed in duplicate. The 2−ΔΔCT was used to determine the relative mRNA expression and normalized using β-actin as a housekeeping gene.

### Cytokine arrays

HUVEC were seeded at approximately 3 × 10^5^ cells per well of a 6-well plate in EGM-2 media. Once monolayers had formed, media was changed to endothelial basal media-2 (EBM-2, Promocell) supplemented with 2% FCS, 1 μg/ml ascorbic acid and 22.5 μg/ml heparin 1 h before treatment with vehicle (H_2_O) or 10 ng/ml IFNγ in a 1.8 ml volume. After 24 h, the cell culture supernatants were collected and immediately frozen in liquid nitrogen. An array of 105 human cytokines was performed using a Proteome Profiler Human XL Cytokine Array Kit (Biotechne, #ARY022B) according to the manufacturer’s instructions. Briefly, the membranes to which capture antibodies were bound in duplicate were blocked for 1 h at room temperature before overnight incubation with cell culture supernatants at 4 °C. Membranes were washed 3 times for 10 min before addition of the detection antibody cocktail for 1 h at room temperature. Washes were repeated and membranes were incubated with streptavidin-HRP for 30 min. Membranes were incubated with ECL reagent (ThermoFisher) and developed using X-ray film with exposure times between 1 and 70 min. The raw signal from each array were quantified by densitometry at an appropriate exposure level and relative cytokine abundance was calculated, normalized to reference spots. The fold-change abundance of all detectable cytokines (relative to the average of duplicate vehicle-treated HUVEC cytokine abundance) was calculated. Cytokines with abundance changes <3-fold or not detected in HUVEC supernatants are displayed in Table S1.

For mouse plasma cytokine arrays, plasma was collected from TET2^fl/fl^ Cre^+ve^ and TET2^fl/fl^ Cre^−ve^ male, tamoxifen-injected mice (n = 8 per group) and pooled equal volumes. An array of 111 mouse cytokines was performed using a Proteome Profiler Mouse XL Cytokine Array Kit (Biotechne, #ARY028) according to the manufacturer’s instructions as described above.

### ELISA

Cell culture supernatants (800 μl) from HUVEC treated with siRNA targeting TET2 or negative control siRNA were collected from confluent 6-well plates, and CXCL9/10/11 expression levels were determined with ELISA kits purchased from R&D systems (Human CXCL9/MIG, #DY392-05; Human CXCL10/IP-10, #DY266-05; Human CXCL11/I-TAC, #DY672). Cell supernatants were diluted 1:8 and the assays were carried out according to the manufacturer’s instructions. The absorbance was read using a microplate reader (Infinite M200 Pro, Tecan) set to 450 nm and wavelength correction set to 540 nm with eight flashes. All samples were assayed in duplicate.

### Hydroxymethylated DNA immunoprecipitation sequencing (hMeDIPseq)

Monolayers of HUVEC were cultured for 25 h in endothelial basal media-2 (EBM-2, Promocell) supplemented with 2% FCS, 1 μg/ml ascorbic acid and 22.5 μg/ml heparin (‘basal media’) or cultured in basal media for 1 h and then treated with 10 ng/ml IFNγ in basal media for 24 h. Genomic DNA was extracted using the Monarch Genomic DNA Purification Kit (#T3010S) method according to the manufacturer’s instructions. hMeDIPseq and initial bioinformatic analysis were carried out by ArrayStar using DNA from vehicle- or IFNγ-treated HUVEC, using the following protocol: DNA samples were fragmented to a size range of ∼200-800 bp with a Diagenode Bioruptor. Approximately 1 μg of fragmented DNA was prepared for Illumina NovaSeq 6000 sequencing as follows: (1) End repair of DNA samples; (2) A single ‘A’ base was added to the 3′ ends; (3) Ligation of Illumina’s genomic adapters to DNA fragments; (4) hMeDIP to enrich hydroxymethylated DNA by anti-5-hydroxymethylcytosine antibody; (5) PCR amplification to enrich precipitated fragments; (6) Size selection of ∼300-900 bp DNA fragments using AMPure XP beads. The completed libraries were quantified by Agilent 2100 Bioanalyzer. The libraries were denatured with 0.1 M NaOH to generate single-stranded DNA molecules, captured on an Illumina flow cell and amplified *in situ*. The libraries were then sequenced on the Illumina NovaSeq 6000 following the NovaSeq 6000 S4 Reagent Kit (300 cycles) protocol. After sequencing images were generated, image analysis and base calling were performed using Off- Line Basecaller software (OLB V1.8). After passing Solexa CHASTITY quality filter, the clean reads were aligned to human genome (UCSC hg19) using Hisat2 software. Aligned reads were used for peak calling, long ncRNA, mRNA and small ncRNA associated hMeDIP enriched regions (peaks) of statistical significance were identified for each sample, using a q-value threshold of 10^−4^ by MACS2 software. Long ncRNA-, mRNA- and small ncRNA-associated hMeDIP enriched regions (peaks) were annotated by the nearest gene using the UCSC hg19 RefSeq database. Long ncRNA-, mRNA- and small ncRNA-associated, differentially-hydroxymethylated regions (DhMRs) within promoter regions, showing statistical significance between two samples/groups, were identified by diffReps software (Cut-off: log2FC ≥ 0.585, *p*-value ≤ 0.001). The hydroxymethylation signal at specific genomic loci was visualized using the UCSC genome browser. Data are publicly available *via* the GEO database, deposited under GSE232280.

### Nanopore sequencing and visualization

HUVEC were cultured to confluency for 1 week in EGM-2 containing 25 mM mannitol in 0.4% gelatin-coated T75 flasks. Media was changed to basal media for 1 h and then continued in the presence or absence of 10 ng/ml IFNγ for 24 h. Genomic DNA was extracted from vehicle- and IFNγ-treated cells using to the Monarch Genomic DNA Purification Kit (#T3010S) according to the manufacturer’s instructions. Prior to making the libraries, samples were firstly fragmented using covaris gtubes at 5000 rpm. Once fragmented, they underwent a combined FFPE repair/end prep step using an end repair module (NEBNext) and 1 μg DNA, followed by a native barcoding ligation step where unique dT-tailed barcodes provided in the native barcoding kit (SQK-NBD114.24, Oxford Nanopore Technologies) were attached to each individual sample. Samples were immediately pooled together in their batches before undergoing the final step of the protocol where sequencing adapters provided in the native barcoding kit were ligated to the cohesive ends of the unique barcodes on samples. Before samples were loaded and sequenced, R10.4.1 flow cells were primed using a priming mix within the provided kit. Libraries were then prepared with the addition of sequencing buffer and library beads before being loaded. The sequencing run set up was using the MinKNOW 24.02.19 software with adaptive sampling integrated. An alignment reference and BED file previously made was uploaded onto the MinKNOW software to ensure this. Sample were then sequenced for 72 h using a PromethION P24 instrument.

The analysis began with POD5 files as the initial input, which were basecalled using Dorado v0.8.1 with the High-Accuracy (HAC) v4.3.0 model, specifically designed for detecting 5mC and 5hmC modifications. Following basecalling, the reads were demultiplexed using Dorado v0.8.1 to separate individual samples based on their barcodes. Each demultiplexed BAM file was then processed separately, ensuring that downstream variant calling was performed on distinct datasets. Variant calling and analysis were conducted using the wf-human-variation pipeline (v2.4.1-g64fe2fb) with the following arguments: -sv --snp --cnv --str --mod --bam_min_coverage 0 --phased --force_strand, enabling the detection of structural variants (SVs), small nucleotide polymorphisms (SNPs), copy number variations (CNVs), short tandem repeats (STRs), and modified bases while enforcing strand specificity and phased analysis. The pipeline produced bedMethyl tables per haplotype, which can be used for differentially methylated region (DMR) analysis. To summarize the modification data, modkit v0.4.1_cec0a0b was used on the haplotagged BAM files, providing a detailed overview of modification signals 5-Methylcytosine (5mC) and 5-Hydroxymethylcytosine (5hmC). Finally, methylation data visualization was performed using MethylArtist v1.3.1, allowing for an in-depth inspection of methylation patterns across regions of interest.

### Data analysis and statistics

Graphing and statistical analysis were performed using GraphPad Prism software (version 9). Data are presented as mean ± standard error of the mean (SEM). A Shapiro–Wilk test was conducted to determine whether data followed a normal distribution. Unpaired t-tests, one-way analysis of variance (ANOVA) or two-way ANOVA tests were used as appropriate. *p* < 0.05 was considered statistically significant.

## Data availability

The datasets generated and analysed during the current study are available in the Gene Expression Omnibus (GEO) repository under accession numbers GSE232888 and GSE232280.

## Supporting information

This article contains supporting information.

References cited in supporting information (30, 81).

## Conflict of interest

The authors declare that they have no conflicts of interest with the contents of this article.
