# Supplementary material for: TET2 has endothelial-specific roles in interferon responses that are dysregulated by hyperglycemia in vitro and in vivo
Source: J Biol Chem. 2025 Jul 25;301(9):110520. doi: 10.1016/j.jbc.2025.110520 (PMC12926064; doi:10.1016/j.jbc.2025.110520)
Supplement: Supporting Figures and Tables [file mmc1.docx]

Supporting information

**TET2 has endothelial-specific roles in interferon responses that are dysregulated by hyperglycaemia *in vitro* and *in vivo***

Hannah L. H. Green^1^, Hashum Sum^1^, Palak Sinha ^1^, Asjad Visnagri^1^, Sang-Hyuck Lee^2^, Anastasia Baffour-Kyei^2^, Hyunah Lee^2^, Konstantinos Theofilatos^1^, Alison C. Brewer^1^*

*^1^School of Cardiovascular and Metabolic Medicine & Sciences, British Heart Foundation Centre of Research Excellence, King’s College London, London, UK*

*^2^ Institute of Psychiatry, Psychology and Neuroscience, King’s College London, London, UK*

Contents:

Table S1

Figures S1-S5

| Cytokine | Fold change vs vehicle | |
| --- | --- | --- |
|  | IFNγ 1 | IFNγ 2 |
| CXCL9 | 58.24 | 193.97 |
| CXCL11 | 23.46 | 20.77 |
| IL-18 Bpa | 18.57 | 20.73 |
| CXCL10 | 9.27 | 8.99 |
| IFNγ | 3.33 | 4.30 |
| Kallikrein 3 | 5.71 | 0.11 |
| MCP-3 | 2.10 | 2.61 |
| IL-10 | 1.38 | 2.46 |
| IL-24 | 1.17 | 1.83 |
| IL-17A | 1.38 | 1.30 |
| MIF | 1.03 | 1.25 |
| MIP-3α | 0.94 | 1.30 |
| CD30 | 1.23 | 1.01 |
| RANTES | 1.15 | 1.04 |
| IL-8 | 1.05 | 1.08 |
| Serpin E1 | -1.01 | -1.04 |
| MCP-1 | -1.07 | -1.23 |
| Thrombospondin-1 | -1.18 | -1.15 |
| PDGF-AB/BB | -1.22 | -1.23 |
| GROα | -1.30 | -1.51 |
| EMMPRIN | -1.66 | -1.27 |
| Endoglin | -1.43 | -1.48 |
| Pentraxin 3 | -1.52 | -1.48 |
| IL-32 | -3.19 | -1.07 |
| Osteopontin 6 | -1.60 | -2.05 |
| GDF-15 | -1.85 | -1.89 |
| Resistin | -2.01 | -2.05 |
| GM-CSF | -1.98 | -2.12 |
| ICAM-1 | -2.33 | -1.83 |
| Dkk-1 | -2.33 | -2.09 |
| PDGF-AA | -2.40 | -2.28 |
| CD31 | -2.68 | -2.61 |
| FGF-19 | -2.47 | -2.92 |
| IL-6 | -3.40 | -2.32 |
| TFF3 | -2.91 | -2.91 |
| ST2 | -2.97 | -2.98 |
| HGF | -4.55 | -2.58 |
| uPAR | -3.61 | -3.38 |
| IGFBP-2 | -2.01 | -29.50 |
| TARC | -3.61 | -3.94 |
| CD40 ligand | -4.24 | -4.75 |
| Vitamin D BP | -6.33 | -6.22 |
| C-Reactive Protein | -4.20 | -12.59 |
| IL-11 | -4.05 | -18.04 |
| G-CSF | -8.68 | -6.50 |
| ENA-78 | -6.73 | -8.88 |
| M-CSF | -5.98 | -11.48 |
| SDF-1α | -6.80 | -21.79 |
| IL-12 p70 | -9.76 | -11.27 |
| Cystatin C | -27.95 | -6.61 |
| TfR | -11.26 | -36.78 |
| EGF | -16.12 | -18.86 |
| Complement Factor D | -17.77 | -87.91 |
| BDNF | -23.31 | -151.79 |
| Angiopoietin-2 | -37.62 | -94.45 |
| IL-1α | -107.87 | -182.76 |

Table S1**:** **Cytokine abundance detected in the supernatant of HUVEC treated with IFN**γ **compared to vehicle-treated HUVEC.** HUVEC were cultured for 24h in the presence or absence of 10ng/ml IFNγ. Media was collected and subjected to a Proteome Profiler Human XL Cytokine Array, detected in duplicate. The raw signal from each array were quantified by densitometry and relative cytokine abundance was calculated, normalised to reference spots. The fold-change abundance of all detectable cytokines in duplicate (relative to the average of duplicate vehicle-treated HUVEC cytokine abundance) is presented in the table. The following cytokines were also included in kit but not detected in HUVEC supernatants: IL-22 TIM-3 VCAM-1 SHBG TGF-α TNF-α VEGF Myeloperoxidase PF4 RAGE RBP-4 Relaxin-2 Leptin LIF Lipocalin-2 3 MIP-1α/MIP-1β MIP-3β MMP-9 IL-19 IL-23 IL-27 IL-31 IL-33 IL-34 IL-4 IL-5 IL-13 IL-15 IL-16 Growth Hormone IGFBP-3 IL-1β IL-1ra IL-2 IL-3 Fas Ligand FGF basic FGF-7 Flt-3 Ligand Chitinase 3-like 1 Cripto-1 DPPIV Adiponectin Apolipoprotein A-I Angiogenin Angiopoietin-1 Complement Component C5/C5a CD14 BAFF.


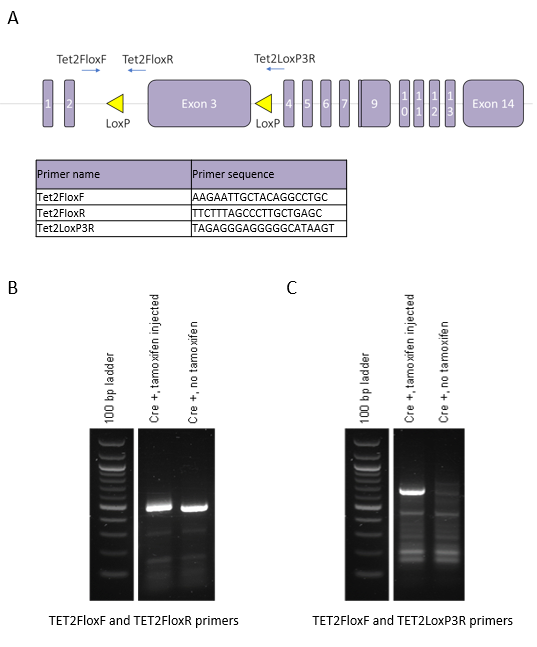


Figure S1: **Confirmation of successful Cre-recombination in mouse lung ECs.** A) Schematic diagram, adapted from [30], showing the location of LoxP sites flanking exon 3 of the TET2 gene. Arrows above indicate the position of primers (in table below) used to identify the presence of the floxed allele and confirm successful Cre recombination. B) CD31+ cells were isolated from the lungs of TET2^fl/fl^ CDH5-CreERT2+ mice. Agarose gel image of PCR products from amplification of CD31+ cell extracts using primers TET2FloxF and TET2FloxR to confirm the presence of the floxed TET2 allele. C) Agarose gel image of PCR products from amplification of CD31+ cell extracts using primers TET2FloxF and TET2LoxP3R to confirm Cre recombination in Cre+ve cells after tamoxifen injection and the absence of Cre recombination in cells from Cre+ve mice without tamoxifen injection. Panels B and C are images taken from a single gel with a common100bp ladder. Thus the ladder is identical in both panels.


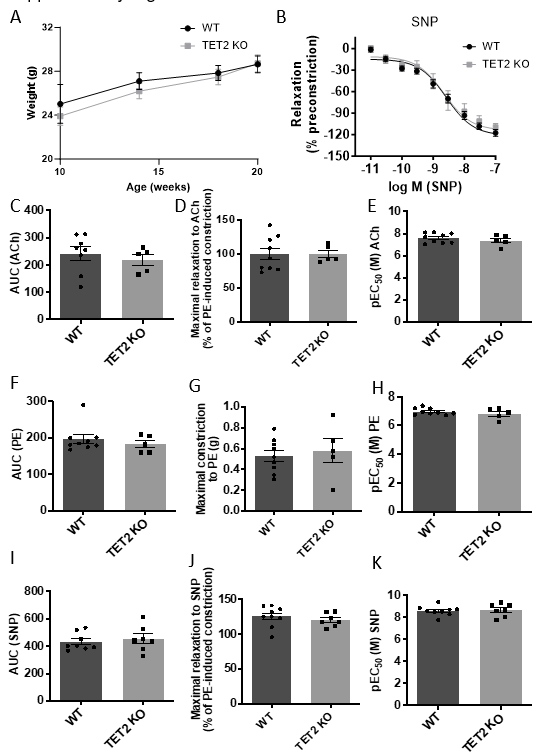


Figure S2: **Body weight and aortic vasoconstrictor/dilator responses in WT and EC-specific TET2 KO mice (20 weeks).** A) Body weight of WT and EC-specific TET2 knockout mice. B) Dose-response curve of aortic relaxation response to 10^-11^ to 10^-5^M concentrations of SNP following pre-constriction with 3x10^-9^M PE in WT and TET2 KO mice. Area under the curve (AUC) of dose-responses of WT and TET2 KO aortae to ACh (C), PE (F) and SNP (I). Maximal percentage relaxation of ACh-treated aortae (D) or SNP-treated aortae (J) from WT and TET2 KO mice. Maximal tension generated upon treatment with PE (G). -log of the half maximal effective concentration (pEC50) of dose response curves to ACh (E), PE (H) and SNP (K) in WT and TET2 KO aortae.


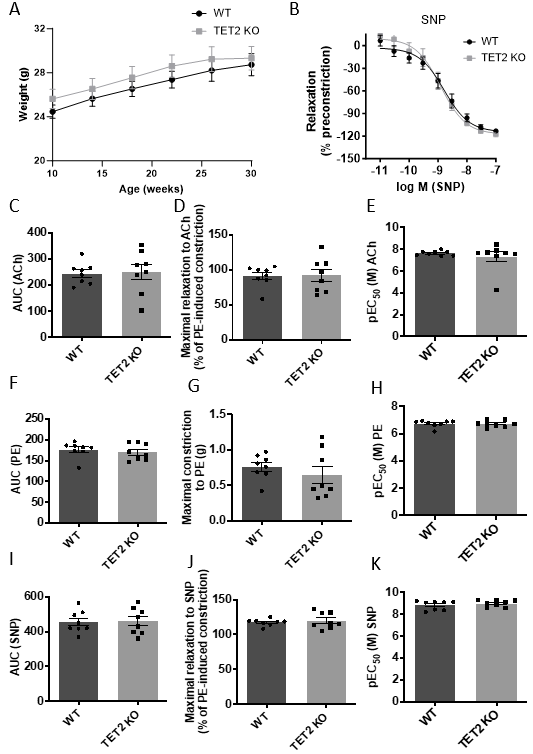


Figure S3: **Body weight and aortic vasoconstrictor/dilator responses in WT and EC-specific TET2 KO mice (30 weeks).** A) Body weight of WT and EC-specific TET2 knockout mice. B) Dose-response curve of aortic relaxation response to 10^-11^ to 10^-5^M concentrations of SNP following pre-constriction with 3x10^-9^M PE in WT and TET2 KO mice. Area under the curve (AUC) of dose-responses of WT and TET2 KO aortae to ACh (C), PE (F) and SNP (I). Maximal percentage relaxation of ACh-treated aortae (D) or SNP-treated aortae (J) from WT and TET2 KO mice. Maximal tension generated upon treatment with PE (G). -log of the half maximal effective concentration (pEC50) of dose response curves to ACh (E), PE (H) and SNP (K) in WT and TET2 KO aortae.


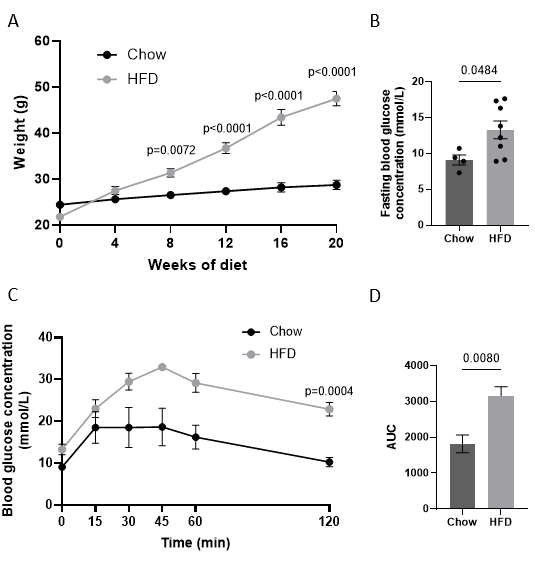


Figure S4: **Induction of obese, diabetic phenotype following high fat diet (HFD) feeding.** Male Cre-ve TET2fl/fl mice were fed a standard chow diet (13% kcal from fat) or a high fat diet (60% kcal from fat) for 20 weeks. A) Body weight of standard chow-fed and HFD-fed mice. B) Blood glucose concentration after 4h fast after 9 weeks of diet. C) Blood glucose concentration during a 120min timecourse following intraperitoneal injection of D-glucose in saline (2g/kg dose). D) Area under the curve of blood glucose concentration. n= 8 mice per group in (A), n=4 Chow and n=8 HFD in (B-D). A Shapiro-Wilk test for normality was performed, followed by 2-way ANOVA.


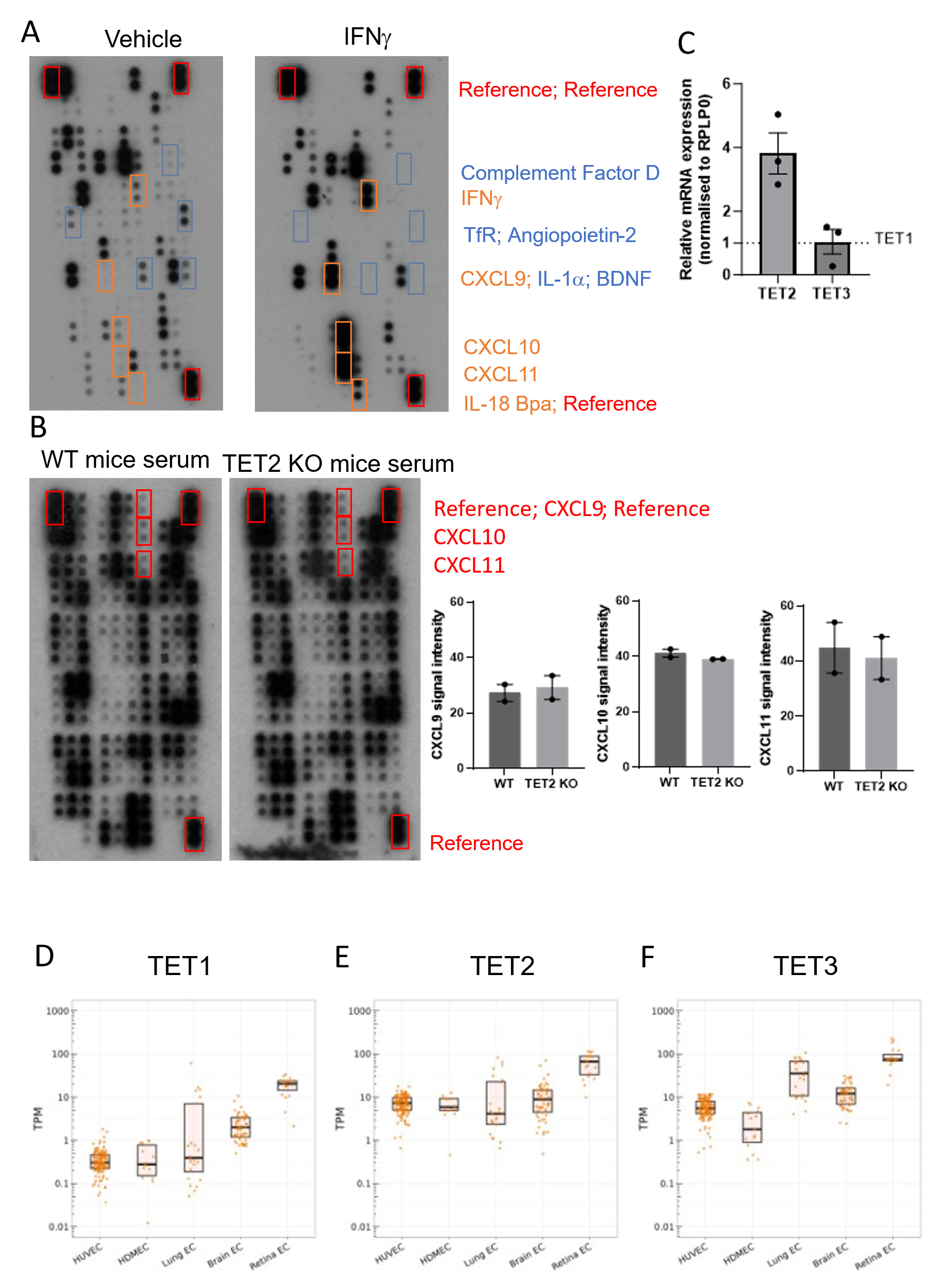


Figure S5: **DNA methylation-regulated expression of CXCR3 ligands and expression of TET1, TET2 and TET3 in ECs.** A) Representative cytokine array from 70min exposure time, highlighting reference spots and the top 5 cytokines with the highest fold-change between supernatants of vehicle-treated and IFNγ-treated HUVEC. B) Array and quantification of abundance of cytokines in pooled plasma from WT and endothelial-specific TET2 KO mice, highlighting reference spots and CXCL9, CXCL10 and CXCL11. C) mRNA expression of TET2 and TET3 in HUVEC relative to TET1, indicated by the dotted line at y=1 (measured by qPCR and normalised to RPLPO as a housekeeping gene). F-H) Number of transcripts per million (TPM) corresponding to TET1 (D), TET2 (E) or TET3 (F) identified in 5 endothelial subtypes (HUVEC, human dermal microvascular ECs, primary mouse lung, brain and retinal ECs) in a compendium of published bulk RNAseq data sets. Data obtained using BulkECexplorer [80].

Supporting information references

30. Moran-Crusio, K., et al., *Tet2 loss leads to increased hematopoietic stem cell self-renewal and myeloid transformation.* Cancer Cell, 2011. **20**(1): p. 11-24.

80. Brash, J.T., et al., *The BulkECexplorer compiles endothelial bulk transcriptomes to predict functional versus leaky transcription.* Nat Cardiovasc Res, 2024. **3**: p. 460-473.
